# The Full Globin Repertoire of Turtles Provides Insights into Vertebrate Globin Evolution and Functions

**DOI:** 10.1093/gbe/evv114

**Published:** 2015-05-15

**Authors:** Kim Schwarze, Abhilasha Singh, Thorsten Burmester

**Affiliations:** Institute of Zoology, Department of Biology, University of Hamburg, Germany

**Keywords:** gene duplication, globins, neuroglobin, oxygen, retina

## Abstract

Globins are small heme proteins that play an important role in oxygen supply, but may also have other functions. Globins offer a unique opportunity to study the functional evolution of genes and proteins. We have characterized the globin repertoire of two different turtle species: the Chinese softshell turtle (*Pelodiscus sinensis*) and the western painted turtle (*Chrysemys picta bellii*). In the genomes of both species, we have identified eight distinct globin types: hemoglobin (Hb), myoglobin, neuroglobin, cytoglobin, globin E, globin X, globin Y, and androglobin. Therefore, along with the coelacanth, turtles are so far the only known vertebrates with a full globin repertoire. This fact allows for the first time a comparative analysis of the expression of all eight globins in a single species. Phylogenetic analysis showed an early divergence of neuroglobin and globin X before the radiation of vertebrates. Among the other globins, cytoglobin diverged first, and there is a close relationship between myoglobin and globin E; the position of globin Y is not resolved. The globin E gene was selectively lost in the green anole, and the genes coding for globin X and globin Y were deleted in chicken. Quantitative real-time reverse transcription polymerase chain reaction experiments revealed that myoglobin, neuroglobin, and globin E are highly expressed with tissue-specific patterns, which are in line with their roles in the oxidative metabolism of the striated muscles, the brain, and the retina, respectively. Histochemical analyses showed high levels of globin E in the pigment epithelium of the eye. Globin E probably has a myoglobin-like role in transporting O_2_ across the pigment epithelium to supply in the metabolically highly active retina.

## Introduction

Globins are small heme proteins that are well known for their ability to bind molecular oxygen (O_2_), but also other gaseous ligands. Globins are widespread in the animal kingdom. As hemoglobins (Hbs) and myoglobins (Mbs), these proteins serve for the transport and storage of O_2_ for respiratory purposes. However, globins also have other functions, and may be involved in the production and decomposition of nitric oxide (NO), the detoxification of reactive oxygen species (ROS), or intracellular signaling (Burmester and Hankeln 2014).

Globins are a classical model system to study the evolution of genes and proteins (Goodman et al. 1987, 1989; Hardison 1996, 1998; Wajcman et al. 2009; Burmester and Hankeln 2014). Vertebrates possess eight distinct globin types that differ in structure, evolutionary history, and probably also function (Burmester and Hankeln 2014). Hb, which serves for the transport of O_2_ in the red blood cells (Dickerson and Geis 1983), and Mb, which facilitates O_2_ supply within the striated muscles (Wittenberg BA and Wittenberg JB 1989), are the best known and thoroughly investigated globins. Since 2000, however, six additional globin types have been discovered, which mostly have poorly defined functions (Burmester and Hankeln 2014). Three of these “novel” globins are widespread among the jawed vertebrates (Gnathostomata): 1) Neuroglobin (Ngb), which is nerve specific (Burmester et al. 2000); 2) cytoglobin (Cygb), which is mainly expressed in fibroblast but also some nerve cells (Kawada et al. 2001; Burmester et al. 2002; Trent and Hargrove 2002); and 3) androglobin (Adgb), which is predominantly expressed in the testes (Hoogewijs et al. 2012). Three other globins have a more restricted taxonomic distribution and phylogenetic analyses suggest that the genes have been secondarily lost in some vertebrate taxa (Hoffmann et al. 2011; Schwarze and Burmester 2013; Schwarze et al. 2014): 1) Globin X (GbX), which is a membrane-bound globin in the nervous system, has not been found in birds and mammals (Roesner et al. 2005; Blank, Wollberg, et al. 2011); 2) globin Y (GbY) has yet poorly defined expression patterns and taxonomic distribution (Fuchs et al. 2006); and 3) globin E (GbE) is an eye-specific globin found in birds, some reptiles, and the coelacanth (Kugelstadt et al. 2004; Hoffmann et al. 2011; Schwarze and Burmester 2013).

The functions of the globins other than Hb and Mb are still poorly understood (Burmester and Hankeln 2014). The high expression levels of GbE in the retina of chicken and similar O_2_-binding kinetics suggest that this protein has an Mb-like function (Blank, Kiger, et al. 2011). Several lines of evidence suggest that Ngb is involved in the oxidative metabolism of the neurons (Hankeln et al. 2005; Burmester and Hankeln 2009). On the one hand, Ngb may enhance local O_2_ supply to the mitochondria, on the other hand, Ngb may protect the neurons from oxidative and other stresses. Cygb may provide O_2_ to specific enzymes (Schmidt et al. 2004), may be involved in the NO metabolism (Avivi et al. 2010; Hundahl et al. 2013), may protect the cells from ROS (Fang et al. 2011; Singh et al. 2014), or may function in signaling pathways (Reeder et al. 2011). GbX harbors N-terminal acylation sites (myristoylation at Gly2 and palmitoylation at Cys3), by which it is anchored in the membrane (Blank, Wollberg, et al. 2011). GbX may be involved in the protection of the membrane lipids or an unknown signaling process (Burmester and Hankeln 2014). As the Adgb is specifically expressed in the testis, a function in spermatogenesis may be assumed (Hoogewijs et al. 2012). For GbY, there is still too little information to speculate about its function.

The first gnathostome vertebrate had eight different types of globins with distinct functions (Burmester and Hankeln 2014). The loss of specific globin genes in some vertebrate lineages may be explained by the fact that its specific function had become unnecessary or that another (globin) gene had taken over its role. Most vertebrates today do not harbor the full globin repertoire (Burmester and Hankeln 2014), hampering a comparative analysis of globin patterns in a single species. The only vertebrate so far with a full set of globin genes was the coelacanth *Latimeria chalumnae* (Schwarze and Burmester 2013), which was therefore referred to as a “globin fossil.” Because of the lack of material, functional studies were not possible with the coelacanth. However, analyses of the genomes of the Chinese softshell turtle (*Pelodiscus sinensis*) (Wang et al. 2013) and the western painted turtle (*Chrysemys picta bellii*) (Shaffer et al. 2013) identified copies of all eight vertebrate globin genes in both species.

Turtles are one of the oldest reptile groups; some turtles show specific adaptations that allow them to survive extreme conditions, for example, hypoxia or even anoxia (Krivoruchko and Storey 2010; Shaffer et al. 2013). During hibernation, many freshwater turtles survive at the bottom of frozen lakes unable to breathe, or in the mud under total anoxic conditions (Ultsch 2006). This tolerance is mainly based on the ability to reduce their energy consumption along with increasing anaerobic adenosine triphosphate (ATP) production (Lutz and Nilsson 1997; Staples and Buck 2009). In anoxia-tolerant turtles, the neuronal activity is reduced by channel and spike arrest during oxygen-deficient periods (Hochachka 1986; Hylland et al. 1997). Nevertheless, the central nervous system and particularly the visual system (Wong-Riley 2010) still have a relatively high ATP turnover.

A main advantage of the turtles is that they are—in contrast to the coelacanth—available for experimental studies. Knowledge of the tissue-specific distribution of the various globins may first provide important clues about globin functions, and second help to understand better how the different globins contribute to the hypoxia adaptation of turtles. Therefore, here we obtained the full globin repertoires of the Chinese softshell turtle and the western painted turtle. We obtained the tissue-specific expression patterns of each globin in both species; we specifically focused on the localization of GbE, for which no data outside the chicken existed (Blank, Kiger, et al. 2011).

## Materials and Methods

### Sequence and Synteny Analyses

The globin genes of the Chinese softshell turtle *P. sinensis* were identified in the genome assembly PelSin_1.0 (Wang et al. 2013) available at ENSEMBL (http://www.ensembl.org, last accessed June 3, 2015) employing the BLAST algorithm (Altschul et al. 1990). The globin repertoire of the western painted turtle *C. picta bellii* along with additional positional information were obtained from the genome assembly ChrPicBel3.0.1 (Shaffer et al. 2013), as available at *Pre*-ENSEMBL (http://pre.ensembl.org, last accessed June 3, 2015). Because of the lack of annotation in *Pre*-ENSEMBL database, the GenBank identifiers (http://www.ncbi.nlm.nih.gov, last accessed June 3, 2015) were used for the painted turtle. In addition to the gene models taken from ENSEMBL and GenBank, gene predictions were carried out manually and with GenScan (http://genes.mit.edu/GENSCAN.html, last accessed June 3, 2015) and Augustus (http://bioinf.uni-greifswald.de/augustus/, last accessed June 3, 2015). Protein sequences were predicted by translation with the web-based tool provided at the ExPASy Molecular Biology Server (http://www.expasy.org, last accessed June 3, 2015). Partial globin sequences were completed by RACE (rapid amplification of cDNA ends) (see below). For gene synteny analysis, the sequences and gene order were obtained from the genome assemblies of the chicken *Gallus gallus* (build 2.1, Annotation release 102), from the green anole *Anolis carolinensis* (AnoCar2.0, Annotation release 101), and from the coelacanth *L. chalumnae* (LatCha1.0) available at the NCBI website (http://www.ncbi.nlm.nih.gov/mapview/, last accessed June 3, 2015).

### Multiple Sequence Alignment and Phylogenetic Reconstructions

To determine the orthology of the turtle globin genes identified in this study, they were added to a recently published data set of vertebrate globin genes (Schwarze et al. 2014). Multiple sequence alignments of the protein sequences were carried out with different algorithms and ranked with MUMSA (Lassmann and Sonnhammer 2005). We used MAFFT with the FFT-NS-i, L-INS-i, and G-INS-i models (Katoh and Toh 2008; Katoh et al. 2009), MUSCLE (Edgar 2004), PROMALS3D (Pei et al. 2008), and T-coffee (Notredame et al. 2000). The MAFFT L-INS-i algorithm received the best MUMSA score and was used for phylogenetic reconstructions. The most appropriate model of amino acid evolution (LG; Le and Gascuel 2008) was selected by ProtTest (Abascal et al. 2005) applying the Akaike Information Criterion. Implementation of phylogenetic analysis was performed with MrBayes 3.2.3 (Huelsenbeck and Ronquist 2001; Ayres et al. 2012) with the LG model of amino acid substitution (Le and Gascuel 2008). Two independent runs with four simultaneous chains and 5,000,000 generations were performed. The trees were sampled every 1,000th generation. The final average standard deviation of split frequencies was <0.01. Convergence was further analyzed by estimating the potential scale reduction factor, which was 1.00. The posterior probabilities were estimated on the final 3,000 trees.

### RNA Extraction and cDNA Cloning

The turtles used in this study were obtained from a pet shop. One Chinese softshell turtle and three western painted turtles, each 2 years old, were used in this study. All animal handling were done in compliance with the guidelines of the German Animal Welfare Act. The animals were sacrificed, tissues were collected and stored in RNAlater (Qiagen, Hilden, Germany) at −20 °C. Total RNA from each tissue sample (brain, eye, muscle, heart, kidney, liver, intestine, lung, and blood) was extracted using peqGOLD Trifast (PEQLAB, Erlangen, Germany) and Crystal RNA Mini Kit (Biolab Products, Gödenstorf, Germany) according to manufacturer’s instructions. Samples were treated with on-column RNase-free DNase (Qiagen) and the integrity of the RNA was assessed by denaturating gel electrophoreses. Reverse transcription (RT) of 750 ng total RNA was performed with the RevertAid H Minus First Strand cDNA Synthesis Kit (Thermo Scientific, Bonn, Germany) with oligo-(dT)_18_-primer according to manufacturer’s instructions. Gene-specific oligonucleotides (supplementary table S1, Supplementary Material online) were used for amplification of selected turtle globin cDNAs. For the painted turtle globins, different oligonucleotides were used to construct either the standard plasmids or for amplification in quantitative real-time RT polymerase chain reaction (qRT-PCR) (see below). The standard plamids were constructed with 400–500 bp fragments of the respective globin. Fragments of 100 bp fragments of each globin were amplified by qRT-PCR (see below). For the softshell turtle, we used the qRT-PCR primers also to construct standard plasmids. The PCR products were cloned into the pGEM-T/JM109 system (Promega, Mannheim, Germany) and sequenced by a commercial service (GATC, Konstanz, Germany). Missing 3′ and 5′ ends of cDNAs were obtained by RACE using the GeneRacer Kit (Invitrogen, Carlsbad, CA) according to the manufacturer’s instructions.

### Quantitative Real-Time Reverse Transcription Polymerase Chain Reaction

The expression of globin messenger RNAs (mRNAs) were estimated by qRT-PCR. We determined the globin mRNA levels from brain, eye, muscle, heart, kidney, liver, intestine, and lung, each from the Chinese softshell turtle and three western painted turtles. Blood subsamples were used from two western painted turtles. The Adgb mRNA expression level was obtained from two western painted turtles. qRT-PCR amplification (40 cycles: 95 °C for 15 s, 60 °C for 15 s, 72 °C for 30 s, detection at last step) was carried out on an ABI 7500 real-time PCR system using the ABI Power SYBR Green master mix (Applied Biosystems, Darmstadt, Germany). Experiments were performed as triplicates in a volume of 20 µl with a final cDNA amount equivalent of 37.5 µg total RNA and 200 nM of each intron-spanning oligonucleotide (supplementary table S1, Supplementary Material online). Negative controls without cDNA were included. Success and specificity of amplification were evaluated using dissociation curve analyzes. We calculated the mRNA copy number with a standard curve method, in which duplicates of recombinant plasmids with known copy number representing each globin cDNA were run using serial dilutions 10-fold (10^7^–10^2^). The samples were normalized according to 1 µg of total RNA.

### SDS-PAGE and Western Blotting

Frozen eye samples of the Chinese softshell turtle and the chicken, respectively, were each homogenized in 10 mM Tris–HCl, pH 7.4, 10 mM NaCl, 5 mM MgCl_2_, 1 mM dithiothreitol (DTT), 1 mM Pefabloc SC protease inhibitor (Carl Roth) and Complete protease inhibitor mix (Roche Applied Science) using the Sonopuls HD2070 Ultrasonic Homogenizer (Bandelin, Berlin, Germany). After 10 min centrifugation at 10,000 × *g* at 4 °C, the protein concentrations of the supernatants were determined using the fluorometric quantification with the Qubit 2.0 (Life Technologies, Darmstadt, Germany). A total of 50 µg protein and 35 µg recombinantly expressed chicken GbE protein were denatured in 65 mM Tris–HCl, pH 6.8, 1% sodium dodecyl sulfate (SDS), 5% β-mercaptoethanol, 10% glycerol at 95°C for 5 min, and separated on a 15% SDS-polyacrylamide gel electrophoresis (PAGE). Proteins in the polyacrylamide gel were visualized with Coomassie Brilliant Blue. The GbE band in the turtle eye protein lane was cut out from the SDS-Gel. Protein sequencing was carried out by static electrospray ionization tandem mass spectrometry at the Department of Clinical Chemistry of the University of Medical Center Hamburg Eppendorf, Germany.

Proteins were transferred onto a 0.22 µm nitrocellulose membrane. Nonspecific binding sites were blocked for 1 h with 2.5% nonfat dry milk in TBS (10 mM Tris–HCl, pH 7.4, 140 mM NaCl) and detection was performed for 2 h at room temperature with affinity-purified polyclonal anti-GbE antibodies (Blank, Kiger, et al. 2011), diluted 1:1,000 in 2.5% milk/TBS. Membranes were washed four times in TBS for 5 min and incubated 1 h with the goat antirabbit antibody coupled with alkaline phosphatase (1:20,000 in TBS; Jackson Immunoresearch Laboratories, West Grove, PA, 111-055-003). The visualization of the protein bands was performed with the NBT/BCIP substrate system.

### In Situ Hybridization

A fresh eye was obtained from an adult female chicken and stored at −80°C before cryosection. The pGEMT-plasmids containing *GbE* and *Ngb* cDNA, respectively, of the Chinese softshell turtle were constructed as described above. The plasmids with chicken *Ngb* and *GbE* cDNA were obtained in an earlier study (Blank, Kiger, et al. 2011). Digoxigenin-labeled antisense and sense mRNA probes were generated using the DIG RNA Labeling Kit (Roche Diagnostics, Mannheim, Germany) with linearized plasmids as templates according to the manufacturer’s instructions.

Frozen eye samples were cut at 16 μm thickness using a Cryostat CM 1950 (Leica, Wetzlar, Germany) and mounted on poly-l-lysine slides (Fisher Scientific, Schwerte, Germany). The sections were fixed on ice for 20 min in 4% paraformaldehyde in phosphate buffered saline (PBS) (140 mM NaCl, 2.7 mM KCl, 8.1 mM Na_2_HPO_4_, 1.5 mM KH_2_PO_4_, pH 6.9) and bleached for 30 min in 3% H_2_O_2_/1% KOH. Sections were neutralized with 1% acetic acid, rinsed twice in 1 × PBS 5 min each, and then acetylated for 10 min in 0.5% acetic anhydride in 0.1 M triethanolamine. After washing in PBS twice for 5 min, slides were dehydrated in a graded ethanol series (70%, 90%, 95%, and 100%, 3 min each) and air dried at room temperature.

Hybridization was performed on coverslipped slides sealed by DPX new (Merck Chemicals, Darmstadt, Germany) with probe mix (400 ng/µl probe, 2.5 mg/ml tRNA, 50 mM DTT) mixed 1:5 in hybridization buffer (50% deionized formamide, 10% dextran sulfate, 1 × Denhardt’s solution, 300 mM NaCl, 10 mM Tris–HCl pH 8.0, 1 mM ethylenediaminetetraacetic acid [EDTA] pH 8.0) at 58 °C, overnight. Posthybridization slides were washed four times in 4 × saline sodium citrate (SSC) (20 × SSC: 3 M NaCl, 0.3 M sodium citrate, pH 7.0), 5 min at room temperature each, before being treated with ribonuclease A (0.18 Kunitz unit/ml, Roth, Karlsruhe, Germany) in 10 mM Tris pH 8.0, 0.5 M NaCl, 0.5 mM EDTA buffer (30 min at 37 °C), rinsed 5 min in 2 × SSC (+1 mM DTT) at room temperature twice, 10 min in 1 × SSC (+1 mM DTT) at RT, 10 min in 0.5 × SSC (+1 mM DTT) at RT, and 30 min in 0.1 × SSC (+1 mM DTT) at 60°C.

Slides were equilibrated 5 min in PBS/0.1% Tween-20 and buffer B (100 mM Tris–HCl, 150 mM NaCl, pH 7.5, 0.5% blocking reagent; Roche Diagnostics) before incubating with alkaline phosphatase-coupled antidigoxigenin antibody (Roche Diagnostics) diluted 1:5,000 in buffer B for 2 h at 37 °C. Two 15 min washes in 100 mM Tris–HCl, 150 mM NaCl, pH 7.5, followed by 15 min incubation in 100 mM Tris–HCl, 100 mM NaCl, 50 mM MgCl_2_, pH 9.5 removed unbound antibodies. The visualization of the probes was performed with the NBT/BCIP substrate system for about 16 h. The reaction was stopped by washing in 100 mM Tris, 1 mM EDTA, pH 7.4 for 15 min. After three washes for 10 min in PBS, the nuclei were stained with Hoechst dye 33258 (0.3 μg/ml; Calbiochem, Darmstadt, Germany) for 15 min at RT. Slides were rinsed 30 s in 95% ethanol; air dried and embedded in 1 × PBS/Glycerin (1:9), covered with a coverslip and fixed by nail polish. Sections were analyzed with an Olympus BX51 research microscope, and the images were combined using Adobe Photoshop CS5 12.0.4.

### Immunofluorescence

The cut, fixed, and bleached eye slices from the adult chicken (described above) were rehydrated in 1 × TBS, and nonspecific binding sites were blocked for 30 min with 1% bovine serum/0.1% Triton X-100/TBS. In immunohistochemistry (IHC), sections were incubated overnight at 4 °C in freshly affinity-purified polyclonal anti-GbE antibodies (Blank, Kiger, et al. 2011), diluted 1:2,500 in 1% bovine serum/0.1% Triton X-100/TBS. For immunofluorescence (IF), the commercial antibodies citrate synthetase (Abcam, Cambridge, UK) and ATP synthase beta (Thermo Scientific) were diluted 1:1,000 and 1: 200, respectively, and incubated overnight at 4 °C. Slides were washed three times in TBS for 10 min. Incubation with the secondary antibody in IHC was performed with a goat antirabbit antibody coupled with alkaline phosphatase (1:20,000 in TBS; Jackson Immunoresearch Laboratories) for 2 h at room temperature. For IF experiments, the secondary antibody used was a donkey anti rabbit F(ab)_2_ fragment coupled to Cy3 (1:2,000 in TBS; Jackson Immunoresearch Laboratories). In IHC, the visualization of the protein was performed with the NBT/BCIP substrate system with 1 µl/ml levamisole (Sigma-Aldrich, Munich, Germany) for 10 min. Slides were rinsed 30 s in 95% ethanol; air dried and embedded in 1 × PBS/glycerol (1:9), covered with a coverslip, and sealed with nail polish. IF slides were washed three times 10 min in PBS and nuclei staining was performed using Hoechst dye 33258 (0.3 mg/ml, Calbiochem) for 15 min at room temperature in the dark. Dried sections were embedded in Mowiol (Calbiochem). Sections were analyzed with an Olympus BX51 research microscope, and the images were combined using Adobe Photoshop CS5 12.0.4.

## Results

### Identification and Analysis of Turtle Globin Genes

**Fig. 1.—:**
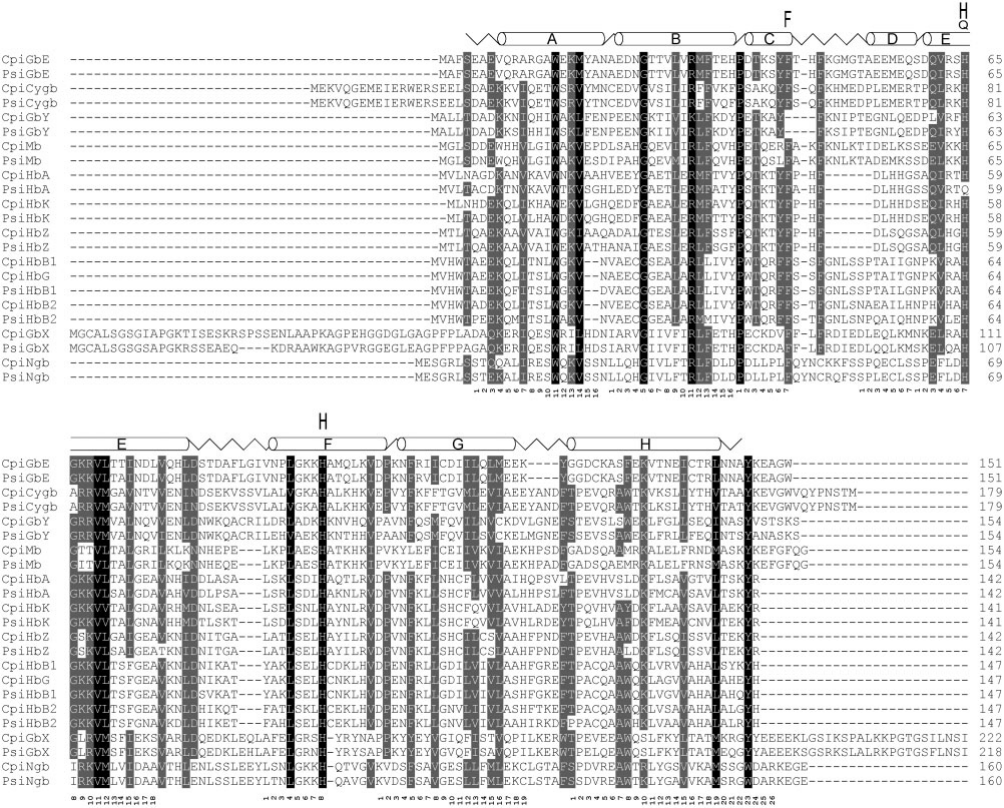
Alignment of globins of the Chinese softshell turtle (*Pelodiscus sinensis*; Psi) and the western painted turtle (*Chrysemys picta bellii*; Cpi). The secondary structure of human Mb is superimposed in the upper row, with α-helices designated A through H, the globin consensus numbering is given below the sequences. Conserved residues are shaded (100% conservation, black; 75%, dark gray). The conserved histidine and phenylalanine residues required for oxygen binding are marked. Note that PsiHbAa harbors a glutamine instead of the histidine at helix position E7.

**Fig. 2.—:**
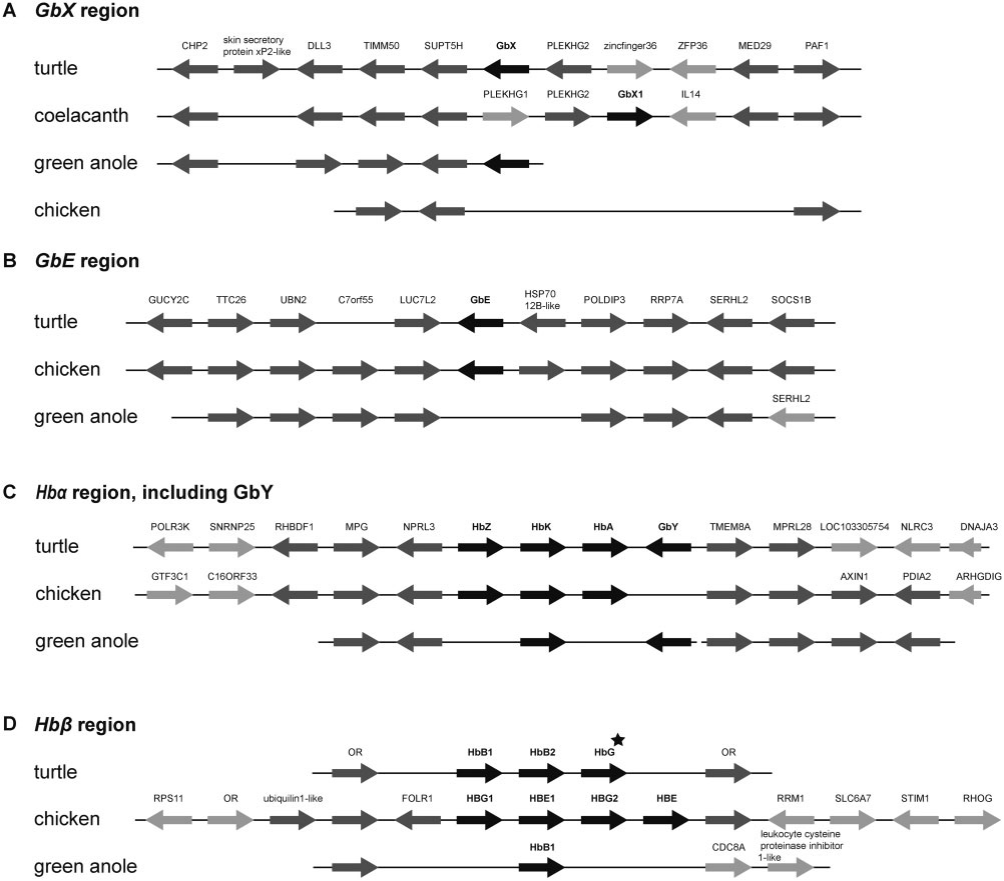
Conserved synteny of the *GbX* (*A*), *GbE* (*B*), *Hbα*/*GbY* (*C*), and *Hbβ* (*D*) chromosomal regions in the genomes of turtle, chicken, and green anole. Globin genes are shown in black; conserved neighboring genes are shaded in dark gray and genes without orthologs in the homologous regions are shaded in light gray. *HbG* (marked with an asterisk) is only present in the painted turtle. Otherwise, the two turtle species have the same gene configuration. Because of the lack of *GbX* in chicken, the coelacanth was included in the analysis. Synteny analyses of the *Mb*, *Cygb*, and *Ngb* chromosomal regions are given in supplementary figure S1, Supplementary Material online.

The globin gene sequences of the Chinese softshell turtle (*P. sinensis*) and the western painted turtle (*C. picta bellii*) were identified in the published genome assemblies (Shaffer et al. 2013; Wang et al. 2013) and with RACE if required. The globin repertoire of the Chinese softshell turtle comprised the full set of vertebrate globins with three *Hb*α and two *Hbβ genes*, and one of each *Mb*, *Ngb*, *GbE*, *GbY*, *GbX*, and *Adgb* gene (fig. 1 and supplementary table S2, Supplementary Material online). The western painted turtle has a higher quality in genome assembly and annotation. It harbors the same gene set and has an additional *Hbβ* gene (figs. 1 and 2*B* and supplementary table S3, Supplementary Material online). Supplementary tables S2 and S3, Supplementary Material online, summarize the names, the lengths, the accession numbers, and the genomic positions of the globins of the softshell turtle and the painted turtle, respectively.

#### Androglobin

Adgb has a modular structure that possesses an N-terminal calpain-like domain, an internal, circularly permuted globin domain, and an IQ calmodulin-binding motif (Hoogewijs et al. 2012). The *Adgb* gene of the softshell turtle is annotated as XM_006111304.1; in the painted turtle, 5 *Adgb* transcripts are annotated with the accession numbers XM_005280422.1 to XM_005280426.1, and code for 1,596 amino acid protein. The protein sequences of the turtle Adgbs display 88.3% identity and 92.6% similarity (supplementary table S4, Supplementary Material online). Synteny analyses of the *Adgb* region shows a high conservation of the neigboring genes *EPM2A-FBXO3O-GRM1-RAB32-ADGB-STXBP5-SAMD5* between the chicken, green anole, and turtle genome sequences (supplementary fig. S1, Supplementary Material online).

#### Neuroglobin

The *Ngb* genes of the softshell turtle and the painted turtle are annotated as XM_006117796.1 and XM_005301643.2, respectively (supplementary tables S2 and S3, Supplementary Material online). The *Ngb* genes have three exons at position B12.2 (i.e*.*, the intron is found between codon positions 2 and 3 in the 12th codon of globin helix B), G7.0, and E11.0, which are typical for Ngb (Burmester et al. 2004). Both turtle globins code for proteins of 160 amino acids, which have 92.5% identity and 96.9% similarity. The genomic organization of *Ngb* in the turtle genomes showed a conserved synteny with the homologous chromosomal regions of chicken and *Anolis* (supplementary fig. S1, Supplementary Material online). In turtles, the analysis of the *Ngb* region was restricted to the 3′-side of the *Ngb* gene due to the fragmentary assembly. The covered region is strictly conserved (supplementary fig. S1, Supplementary Material online).

#### Globin X

In the softshell turtle, no *GbX* gene was annotated or predicted, and therefore identified with Augustus on contig JH211032 at the position 890205-873421. The *GbX* of the painted turtle was misannotated as cytoglobin-1-like (XM_005293187.1). Like the *GbX* genes of amphibians and fishes (Roesner et al. 2005; Blank, Wollberg, et al. 2011), both *GbX* genes of the turtles harbor four introns at positions B12.2, G7.0, H10.0, and E10.2. For synteny analysis of the *GbX* region, we included the *GbX1* contig of the coelacanth. Despite the lack of *GbX* in chicken, the gene synteny in the homologous regions is partially conserved (fig. 2*A*). The comparison of the turtle, coelacanth *GbX1,* and *Anolis GbX* contigs shows a conservation of the neighboring genes, *CHP2-DLL3-TIMM50-SUPT5H-GbX-PLEKHG-MED29-PAF1.* In the coelacanth, the orientation of the region with the genes *GbX* and *PLEKHG* is inversed. The synteny analysis of the globin genes verified the orthology of the identified turtle globin genes to the chicken, coelacanth, and *Anolis* (fig. 2*A*) (Opazo, Lee, et al. 2015).

#### Cytoglobin

There are three transcript predictions for *P. sinensis Cygb* (XM_006136484–XM_006136486) in the databases, which differ in their 3′ coding exon. However, sequence comparisons with other Cygb proteins showed that probably only transcript variant X3 (XM_006136486.1) is correct. In the painted turtle genome, six splice variants are annotated (XM_008163872, XM_005297538, XM_008163873, XM_005297539, XM_005297540, and XM_008163874), with—based on the comparison with the orthologous sequences—variant X5 (XM_005297540.2) probably being correct. Cygb is the most highly conserved globin, displaying 97.8% identity between the two turtles, and 84.9% and 84.4% identity with the chicken Cygb (supplementary table S4, Supplementary Material online). The genomic organization of *Cygb* is conserved in the homologous regions of chicken and *Anolis* and shows that *Cygb* is located between the genes *PRPSAP1-SPHK1-UBE20-AANAT-RHBDF2* and *ST6GALNAC-MXRA7-JMJD6-METTL23* (supplementary fig. S1, Supplementary Material online).

#### Myoglobin

The *Mb* genes of the softshell turtle and the painted turtle are annotated as ENSPIG00000008494 and XM_005300826.2, and XM_005301643.2, respectively (supplementary tables S2 and S3, Supplementary Material online). The coding sequences of the turtle *Mb* genes each translate into proteins of 154 amino acids. The Mb proteins of the turtles share 88.3% of their amino acids and have 92.9% similarity (supplementary table S4, Supplementary Material online). Comparison with the chicken Mb showed 75.3% and 76.6% identity on the protein level. The *Mb* chromosomal region shows the syntenic gene order *HMGXB4-TOM1-HMOX1-MCM5-RASD2-Mb-(APOL3-like)-RBFOX2-MYH9-TXN2-FOXRED2* (supplementary fig. S1, Supplementary Material online), which is conserved among amniotes (Schwarze and Burmester 2013).

#### Globin E

The *GbE* gene (ENSPSIG00000017186) resides on contig JH209952 of the softshell turtle genome and codes for a protein with 151 amino acids. In the painted turtle genome, the *GbE* was misannotated as *cytoglobin-1-like* (XM_005293060.2). The translation of the gene results in 151 amino acid protein. The two turtle GbE proteins display 96.0% identity and 98.7% similarity. Comparison with chicken GbE resulted in identity scores of 85.4%. Evaluation of the *GbE* gene synteny shows the gene order of *LUC7L-GbE* (fig. 2*B*), which is also conserved in the chicken and the coelacanth (Schwarze and Burmester 2013). In the *Anolis* genome, the homologous region is present, although *Anolis* has lost the *GbE* gene (fig. 2*B*). The absence of *GbE* of *Anolis* was verified by amplification and sequencing of the genomic region between the *LUC7L* and *POLDIP3* by PCR.

#### The Hemoglobin Genes

The three *Hb*α genes of the softshell turtle reside on a single contig (JH209131; fig. 2*C* and supplementary table S2, Supplementary Material online). *HbZ* is annotated as ENSPSIG00000012161. Exons 1 and 2 of *HbAd* are annotated as *HbM* (ENSPSIG00000012157); the third exon was retrieved by RACE. Two additional globin gene fragments (ENSPSIG00000011874 and ENSPSIG00000011856), each having the first and second exons, were annotated on the same contig. Completion of the sequences by RACE illustrated that these two sequences, including the untranslated regions, were identical, suggesting either a recent gene duplication or an assembly error. This sequence was identified as *HbAa.* Notably, *HbAα* possesses a glutamine instead of the distal histidine at position E7 (fig. 1). The two *Hbβ* genes (*HbB1* [ENSPSIG00000004624] and *HbB2* [ENSPSIG00000005077]) are located on contig JH210967.

**Fig. 3.—:**
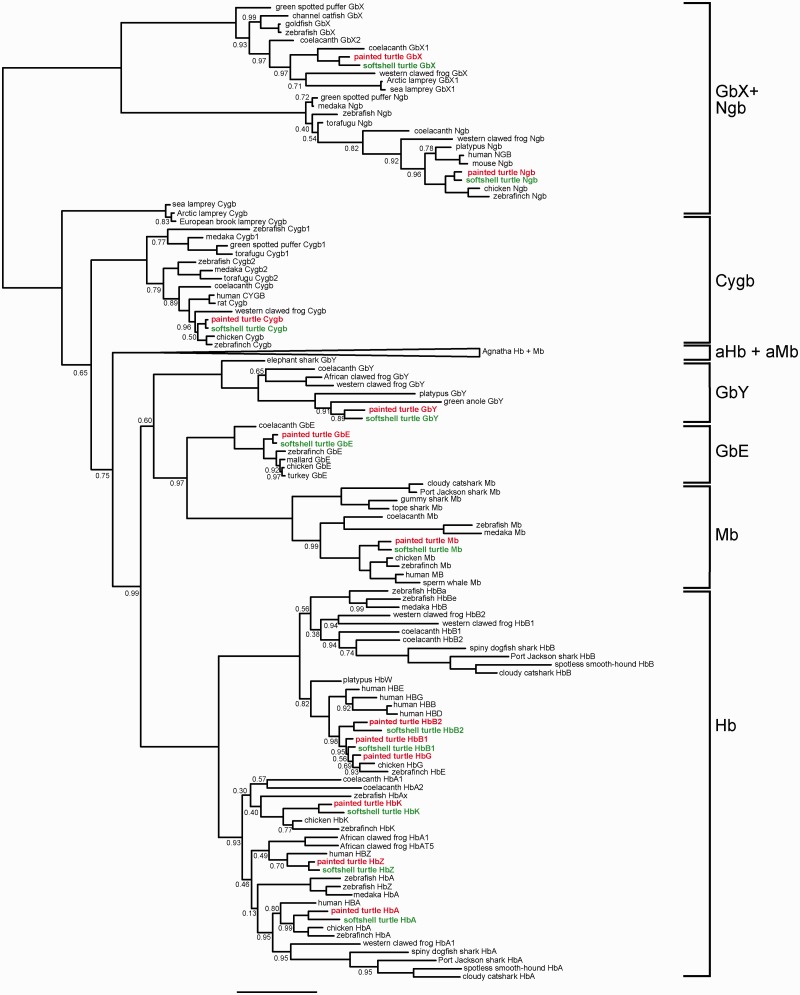
Bayesian phylogenetic tree of vertebrate globins. The Chinese softshell turtle globins are green; the globins of the western painted turtle are red. The numbers at the nodes correspond to the posterior probabilities. The nodes without numbers are supported by 1.0 Bayesian posterior probability. The bar represents 0.5 PAM distance. The common names of the species are given (Schwarze et al. 2014). See supplementary table S5, Supplementary Material online, for further information. The full tree without collapsed branches is given in supplementary figure S2, Supplementary Material online.

The three *Hb*α genes of the painted turtle reside on a single contig (JH584800) in head-to-tail orientation (fig. 2*C* and supplementary table S3, Supplementary Material online). When compared with the putative orthologs in the softshell turtle, HbZ was found to be the highest conserved Hbα (91.5% identity) (supplementary table S4, Supplementary Material online). The HbAd and HbAa of the two turtles share 89.4% and 86.6%, respectively, of the amino acids. All analyzed *Hbα* have 1:1 orthologs in the chicken genome, which display between 82.4% and 90.1% identity (supplementary table S4, Supplementary Material online). In contrast to the softshell turtle, the painted turtle possesses three *Hbβ* genes. Phylogenetic analysis and sequence comparison revealed that *HbB1* and *HbB2* have orthologs in both turtle species, but the softshell turtle lost the ortholog of *HbG* (or it is not represented in the current assembly). Although the *HbB1* and *HbB2* genes have orthologs in chicken, *HbG* is duplicated in chicken and, therefore, there is no 1:1 ortholog to the turtle *HbG* (figs. 2*D* and 3). The turtle and chicken *Hbβ* genes have no 1:1 orthologs in the human genome (Hoffmann et al. 2010) (fig. 3). The *Hb* genes of the turtle are separated on two different clusters (fig. 2*C* and *D*). Gene synteny analyses of the *Hbα* region identified *MPG-NPRL3* and *(GbY)-TMEM8a-MPRL28* as the neighboring genes. The gene order is highly conserved among vertebrates (fig. 2*C*). The numbers of *Hb* genes may differ between closely related species, which is due to a high rate of lineage-specific gene duplication (Hoffmann et al. 2008) (fig. 3). The *Hbβ* genes are embedded in a cluster of olfactory receptor genes (OR) (fig. 2*D*).

#### Globin Y

The *GbY* gene of the softshell turtle (XM_006124603.1) was identified on contig JH209131. In Ensembl, the exon–intron boundaries of the genes (ENSPSIG00000011829) are misannotated. The *GbY* gene of the painted turtle (XM_005306194.2) resides on contig JH584800. In both species, the current annotation (cytoglobin-1-like) is incorrect. The predicted proteins are 80.5% identical (91.6% similarity). In both genomes, *GbY* is adjacent to the *Hbα*-cluster (fig. 2*C*), as it was found in platypus, the green anole, *Xenopus,* and the coelacanth (Hoffmann et al. 2010, 2011; Schwarze and Burmester 2013).

### Phylogeny of Turtle and Other Vertebrate Globins

The amino acid sequences of 163 vertebrate globins were used to construct a multiple sequence alignment (supplementary table S5, Supplementary Material online). A Bayesian analysis with the LG model was used and the resulting tree is displayed in figure 3. The full tree without collapsed branches is given in supplementary figure S2, Supplementary Material online. Adgb was excluded from the analysis because of its highly divergent sequence (Hoogewijs et al. 2012). Ngb and GbX proteins were assumed as the outgroup because they split from the clade that includes the other globins before the divergence of Protostomia and Deuterostomia (Roesner et al. 2005; Blank and Burmester 2012; Hoffmann, Opazo, Storz 2012). We found the Cygb proteins as a paraphyletic assemblage at the base of the other globin types, with the agnathan Cygbs diverging first. However, the support was low (0.65 Bayesian posterior probability [PP]). The next clade is formed by the agnathan Hbs and Mbs (aHb and aMb) (cf. Schwarze et al. 2014). The clade that includes vertebrate Hbα and β, Mb, GbE, and GbY received high support (0.99 PP). Like in other recent analyses (Hoffmann, Opazo, Hoogewijs, et al. 2012; Hoffmann, Opazo, Storz 2012; Schwarze and Burmester 2013; Burmester and Hankeln 2014; Schwarze et al. 2014; Opazo, Lee, et al. 2015), two monophyletic clades that include α- and β-Hb chains on the one hand, and GbE and Mb on the other hand received high support. In this analysis, GbY was found as sister group of the clade comprising GbE and Mb, but the support value was low (0.60 PP).

### Expression Pattern of Globins in Turtle Tissues

We quantified the mRNA levels of *Mb*, *Ngb*, *Cygb*, *GbE*, *GbY*, *GbX*, and *Adgb* by means of qRT-PCR in brain, eye, muscle, heart, kidney, liver, intestine, lung, and blood of the Chinese softshell turtle (*P. sinensis*) and the western painted turtle (*C. picta bellii*). The mRNA levels were normalized according to the total RNA content used for cDNA synthesis and the standard curve method was used to calculate absolute copy numbers.

**Fig. 4.—:**
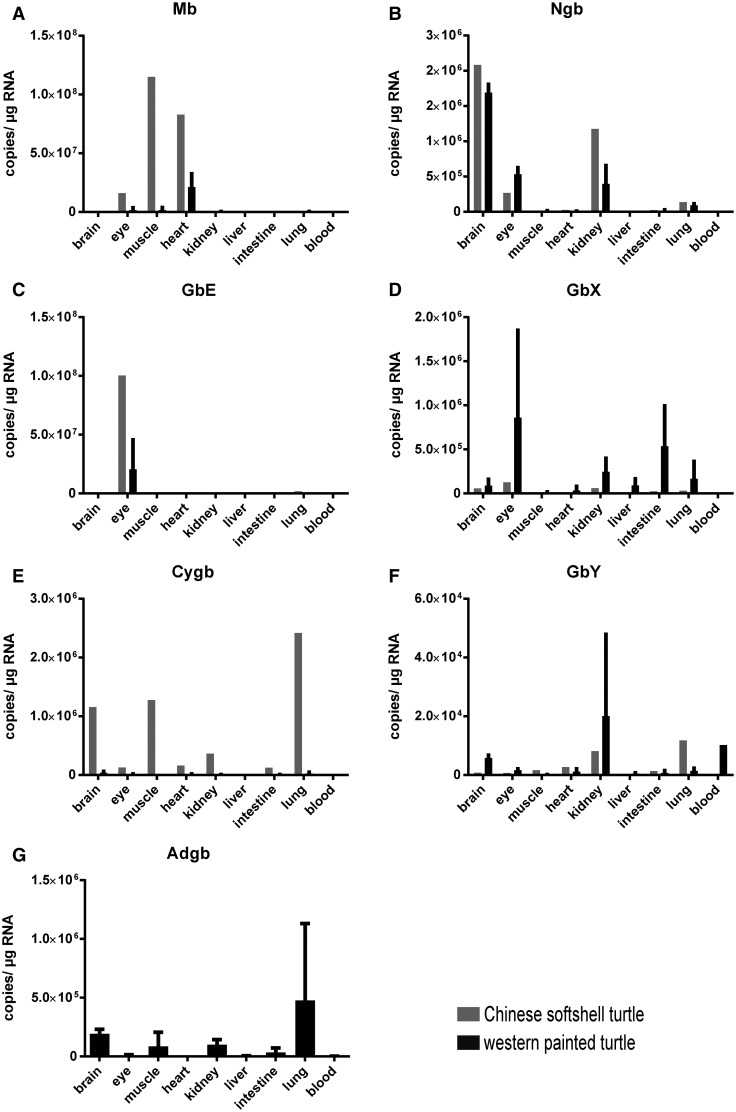
Quantification of mRNA levels of all vertebrate globins (except Hb) in different tissues of the Chinese softshell turtle (gray) and the western painted turtle (black). Absolute copy numbers of mRNA were obtained using qRT-PCR experiments. *Mb* (*A*), *Ngb* (*B*), and *GbE* (*C*) showed a tissue-specific expression pattern in striated muscle, neuronal tissues + kidney and eye, respectively. *GbX* (*D*), *Cygb* (*E*), and *GbY* (*F*) showed a widespread expression in different tissues with lower levels. *Adgb* (*G*), which was only tested in western painted turtle, is expressed in all tissues analyzed. Logarithmic scaled figures are given in supplementary fig. S3, Supplementary Material online.

Evaluation of the globin mRNA levels revealed characteristic expression patterns. In both species, *Mb*, *Ngb*, and *GbE* showed high mRNA levels in specific tissues, whereas *Cygb*, *GbY*, and *GbX* showed a low to moderate expression in a broad range of tissues. In the softshell turtle, *Mb* is highly expressed in muscle and heart tissues with 10^8^ copies per µg total RNA, followed by the amount of 10^7^ copies per µg RNA measured in the eye (fig. 4*A*). In other tissues, the *Mb* expression ranged between 10^1^ and 10^5^ copies per µg RNA. A very similar pattern of *Mb* expression was found in painted turtle, with, however, slightly lower *Mb* mRNA levels (fig. 4*A*). As expected, we detected very high *Ngb* mRNA levels in the brain (∼2 × 10^6^ copies per µg RNA); however, *Ngb* was also moderately expressed in the eye and the kidney (10^5^), but low in the lung in both species (fig. 4*B*). *GbE* showed the most distinct tissue specificity (fig. 4*C*). We measured a very high *GbE* content with 10^8^ copies per µg RNA in the eye of the softshell turtle and 2 × 10^7^ in the painted turtle, respectively. In all other tissues, the *GbE* mRNA levels were at least 1,000-fold lower.

*Cygb*, *GbY*, and *GbX* showed little tissue specificity. However, *GbX* showed a slightly higher expression in the eye than in other tissues (fig. 4*D*). *Cygb* showed consistently low to moderate mRNA level in all tissues in both turtle species (fig. 4*E*). The mRNA levels of *GbY* were not higher than ∼10^4^ copies per µg RNA in any of the analyzed tissues (fig. 4*F*). For a detailed view on low expressed globins, see supplementary figure S3, Supplementary Material online. *Adgb* expression was examined in two biological replicates of the western painted turtle. Low levels of *Adgb* mRNA were detected in all tissues, with being the highest in the lung (fig. 4*G*). However, testis tissue, which is the expected main site of *Adgb* expression, was not available in our studies.

### Localization of GbE in Turtle and Chicken Eye

**Fig. 5.—:**
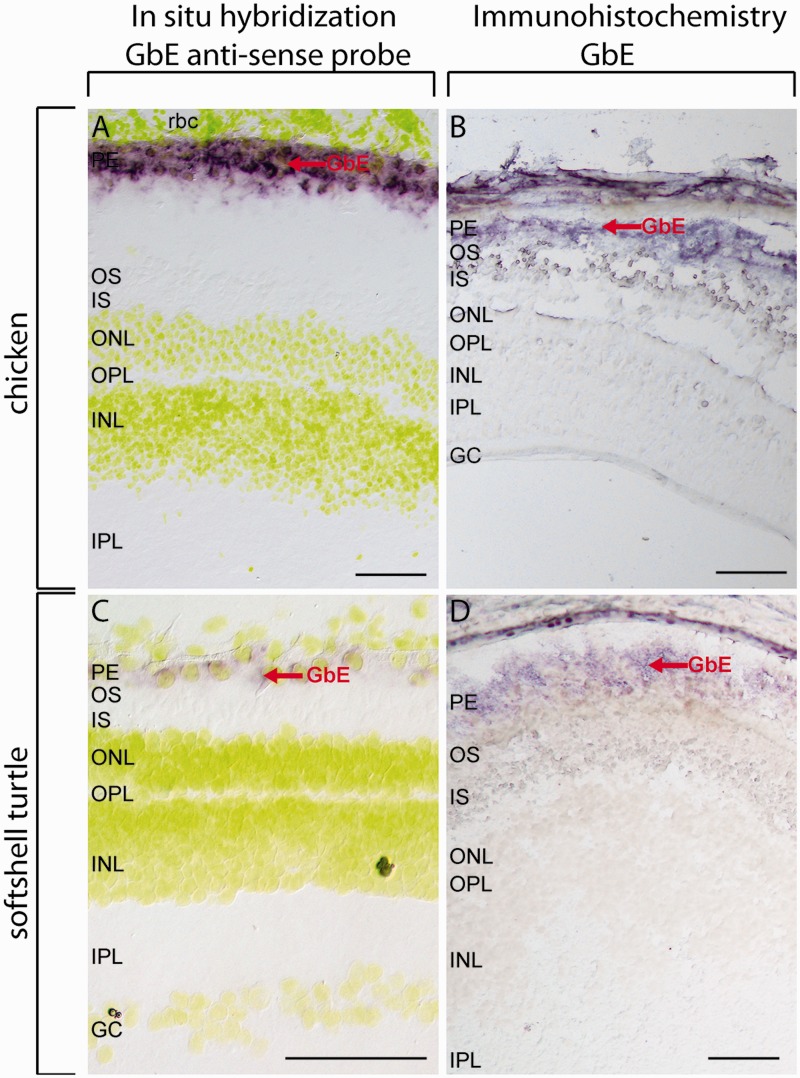
Localization of *GbE* in the retina of softshell turtle and chicken. ISH was carried out with a species-specific antisense probe to detect the *GbE* mRNA (*A*, *C*); for IHC, a specific GbE-peptide antibody was used to detect the GbE protein (*B*, *D*). Both *GbE* mRNA and protein were detected in retinal PE, indicated by red arrows. In ISH, the nuclei were stained with Hoechst dye 33258, shown in yellow. Negative controls with sense probes (ISH) and omitted first antibody (IHC) are shown in supplementary fig. S4, Supplementary Material online. Scale bar = 100 µm. PE: pigment epithelium, OS: outer segments of the photoreceptor cells, IS: inner segments of the photoreceptor cells, ONL: outer nuclear layer, INL: inner nuclear layer, IPL: inner plexiform layer, GC: ganglion cells.

**Fig. 6.—:**
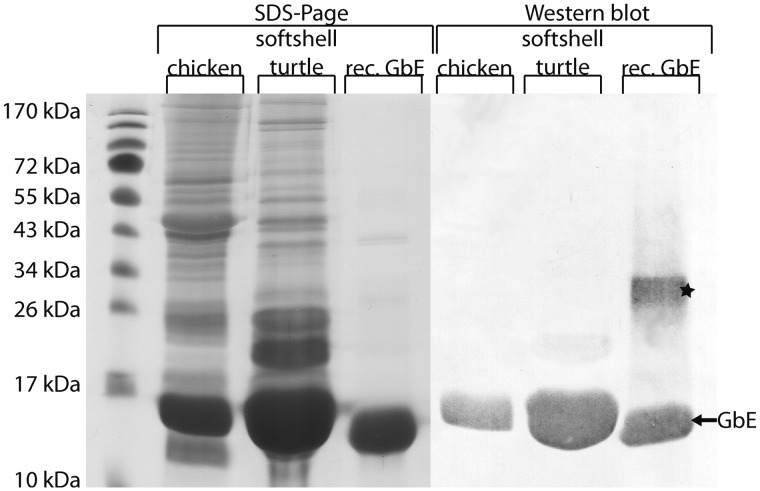
SDS-PAGE and western blot analyses of GbE. Total lysates of chicken and softshell turtle eye and recombinant expressed GbE were separated on SDS-PAGE and stained with Coomassie Brilliant Blue R. In western blot analyses, only the GbE was detected with specific anti-GbE antibody, indicated by an arrow. An asterisk marks the dimer artifact of recombinant GbE.

The distribution of GbE protein and mRNA was characterized in cryosections of the eyes of Chinese softshell turtle and chicken (fig. 5). For immunostaining, we used affinity-purified polyclonal anti-GbE antibodies (Blank, Kiger, et al. 2011). The specificity of the antibodies was checked by SDS-PAGE and western blotting employing whole eye protein extracts along with recombinantly expressed chicken GbE as control. The antibody showed high specificity for GbE in western blot (fig. 6). A second band at ∼30 kDa is likely due to the dimerization of the GbE, which has frequently observed for globins in SDS-PAGE (Schmidt et al. 2003). The predominant band at 15 kDa in the turtle eye was partially sequenced de novo with mass spectrometric methods. The identified peptide (ADAEDNGTTVLVR) corresponds to positions 20–32 of the GbE protein; an exchange of an asparagine to an aspartic acid at position 21 is probably due to deamination during processing.

GbE mRNA and protein were localized in retinal pigment epithelium (RPE) in chicken and turtle retina (fig. 5). The ISH showed the mRNA distribution around the yellow stained nuclei (fig. 5*A* and *C*). Negative controls, in which the primary antibody and sense probe, respectively, were omitted, showed no staining (supplementary fig. S4, Supplementary Material online). In none of the experiments, GbE localization could be detected in outer segments of the photoreceptor cells, as it was previously observed in the chicken retina (Blank, Kiger, et al. 2011).

### Distribution of Mitochondria in Vascular and Avascular Retinae

**Fig. 7.—:**
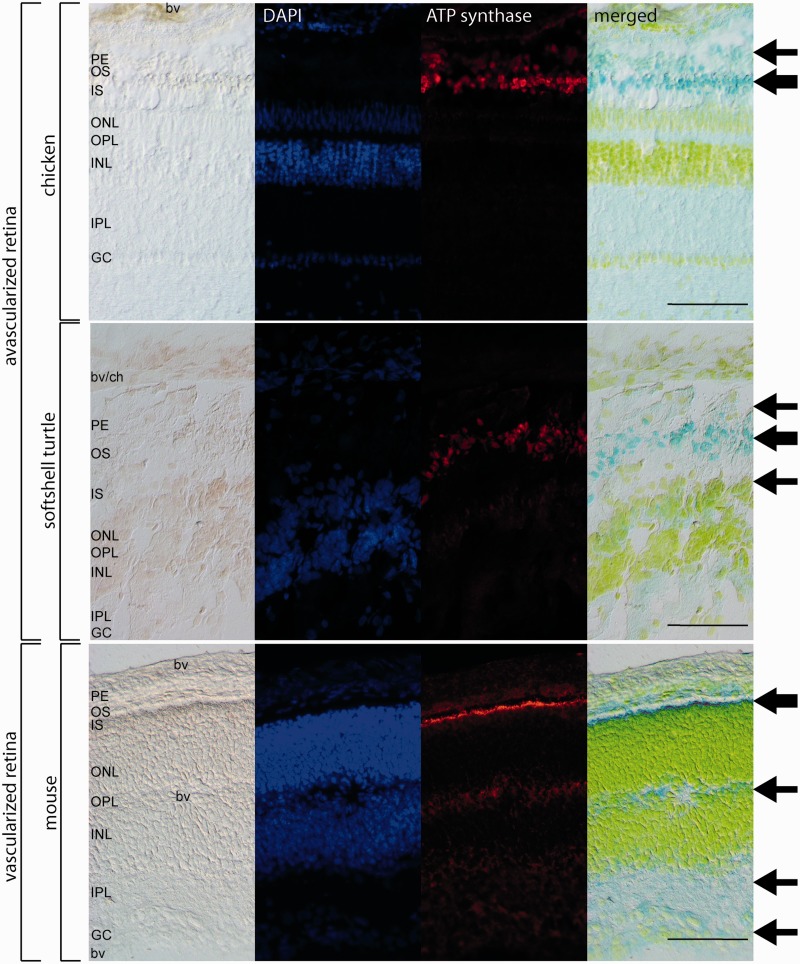
Immunofluorescence of ATP synthase beta in avascular retinae of chicken and turtle and the vascularized retina of the mouse. The intensity of the staining is reflected by the thickness of the arrows in the different retinal layers. In chicken, the mitochondria are stained in the photoreceptor cells (OS, IS) and pigment epithelium (PE). In the softshell turtle, there is also weak staining of the outer nerve layer (ONL). The vascularized retina of the mouse showed mitochondria in the photoreceptor cells (OS, IS), the plexiform layers (IPL, OPL) and the ganglion cell layer (GC). Negative controls with omitted first antibodies are shown in supplementary figure 6, Supplementary Material online. Scale bar = 100 µm. For abbreviations, see figure 5.

To reveal the localization of high densities of mitochondria and thus high oxygen consumption rates, avascular (turtle and chicken) and vascular (mouse) retinae were incubated with antibodies against the mitochondrial enzymes ATP synthase beta (fig. 7) and citrate synthetase (supplementary fig. S5, Supplementary Material online). IF in the avascular retinae of turtle and chicken revealed that mitochondria are mostly localized in the inner and outer segments of the photoreceptor cells, as well as, with lower intensities, in the pigment epithelium and the ganglion cell layer. The retina of the softshell turtle showed additionally a weak staining of the outer plexiform layer with both mitochondrial markers (fig. 7). In the vascular retina of the mouse, the mitochondria are mostly located in the inner segments of the photoreceptor cells, which show a very strong IF signal. Somewhat weaker signals were also detected in the outer segments of the photoreceptor cells, the plexiform layers, and the ganglion cell layer (fig. 7). Negative controls, in which the primary antibodies were omitted, showed no staining (supplementary fig. S6, Supplementary Material online). These different patterns of mitochondria localization match with previous reports of vascular mouse and avascular guinea pig retinas (Bentmann et al. 2005; Stone et al. 2008).

## Discussion

### Turtles Have Retained the Full Vertebrate Globin Repertoire

**Fig. 8.—:**
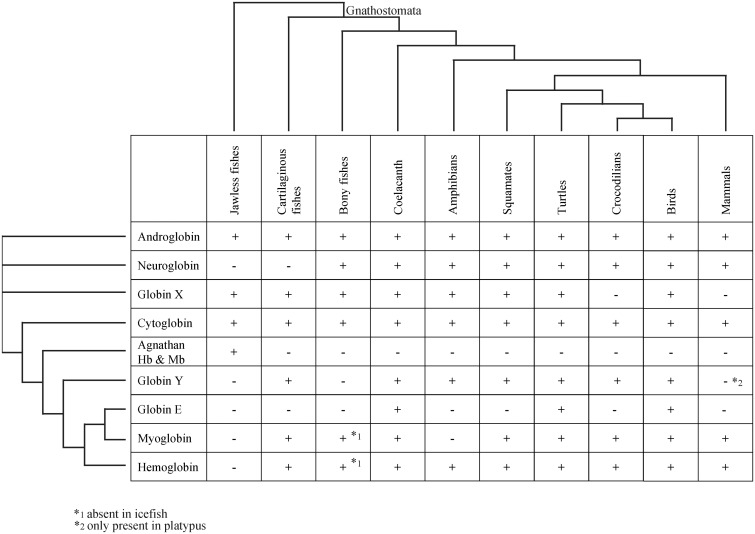
Distribution of globins among the vertebrate tree of life. *1. Absent in some icefish species (Sidell et al. 1997; di Prisco et al. 2002). *2 only in platypus (Hardison 2008).

The distribution of globins among gnathostome vertebrates shows a patchy pattern (fig. 8), which suggests that multiple independent losses of globin genes occurred during the evolution of most taxa (Hoffmann et al. 2011; Storz et al. 2011; Burmester and Hankeln 2014). The loss of certain globin genes may be partly explained by physiological changes that rendered the function of specific globins unnecessary. Other explanations for this pattern may include random changes of the genomes. When we ignore paraphyletic Hb and GbX isoforms (Dickerson and Geis 1983; Opazo, Hoffmann et al. 2015; Opazo, Lee, et al. 2015), the turtles (this study) and the coelacanth (Schwarze and Burmester 2013) are the only known vertebrates with all eight types of gnathostome globins. Notably, both turtles and the coelacanth are morphologically highly conserved taxa that have changed little during the Neozoic period. Furthermore, analyses of the coelacanth and the turtle genomes revealed comparably slow molecular evolution rates (Amemiya et al. 2013; Shaffer et al. 2013). Of course it is difficult to trace the physiological changes of these lineages throughout evolution; however, they did not affect the globin genes and thus the full set of globins had been inherited from the last common ancestor.

Notably, birds and crocodiles do not possess the full globin repertoire. Database searches revealed that birds neither have GbX nor GbY, and that crocodiles and the green anole does not harbor a *GbE* gene (figs. 2*C* and 8). Gene synteny analysis suggest that at least in the anole, the *GbE* gene was selectively lost, rather than being the result of large genome rearrangements. The situation in snakes remains to be thoroughly investigated, with GbY being possibly absent. In any case, the loss of GbX and GbY in birds is well documented and requires explanation. It is noteworthy that both globins are also missing in placental mammals, suggesting that the convergent loss of both globins may be related to their endothermic lifestyle, which provides a constant inner environment, which in turn might render their specific functions unnecessary (Burmester and Hankeln 2014). However, GbX is also absent in crocodiles, and GbY is missing in some fishes, which requires alternative explanations for their loss in certain species.

### Tissue-Specific Expression Provides Clues for Globin Functions

From the perspective of gene expression patterns, there are apparently two types of globins: First, those that are highly expressed in certain tissues (fig. 4); this category includes Hb, which accumulates to high concentrations in the erythrocytes, Mb, which is most highly expressed in the striated muscles, GbE, which is largely restricted to the retina, and Ngb, which is highly expressed in the brain. This indicates that the specific needs of these tissues require high concentrations of the respective globins. There is little doubt that the main functions of Hb and Mb are associated with the oxidative metabolism (Dickerson and Geis 1983; Wittenberg 2003; Burmester and Hankeln 2014). The high concentration of GbE in the retina may easily be associated with the high O_2_ demand of this tissue (see below). The function of Ngb is less well defined (Hankeln et al. 2005; Burmester and Hankeln 2009). However, the relatively high expression in the brain of turtles (2 × 10^6^ copies per µg total RNA) corroborates views that Ngb contributes to O_2_ supply, at least in this taxon. It is tempting to assume that the high levels of Ngb contribute to the remarkable hypoxia-tolerance of turtles (Milton et al. 2006; Bickler and Buck 2007; Larson et al. 2014; Krivoruchko and Storey 2015). Furthermore, Milton et al. (2006) showed an increase in Ngb mRNA expression during hypoxia and post-anoxia-reoxygenation in the red-eared slider *Trachemys scripta*. The expression data further corroborate the hypothesis (Burmester and Hankeln 2014) that there is a positive correlation of Ngb levels and hypoxia tolerance in cross-species comparisons (Roesner et al. 2008; Avivi et al. 2010; Schneuer et al. 2012).

Second, Cygb, GbX, and GbY are expressed in a broad range of tissues (fig. 4). This does not necessarily mean that they are expressed in all cells. For example, the broad distribution of Cygb may be explained by its expression in fibroblasts that form the connective tissue, along with an expression in peripheral neurons, as it has been identified in mice (Schmidt et al. 2004). The expression pattern of GbX in the turtles largely agrees with that observed in the frog (*Xenopus* sp.), where moderate mRNA levels were found in the eye, and weak expression also in gut, ovary, brain, heart, and kidney, but not in muscle (Fuchs et al. 2006). This pattern might be explained by the expression of GbX in neurons that may be associated with the sensory system, as observed in zebrafish *Danio rerio* (Blank, Wollberg, et al. 2011). It should be noted, however, that the *GbX* genes of the turtles and *Xenopus* sp. are not orthologous (Opazo, Lee, et al. 2015).

We further observed Mb and Ngb mRNA above background levels outside their main expression domains. Notable amounts of *Mb* mRNA were also found in the eye, the lung, and the kidney (supplementary fig. S3, Supplementary Material online); *Ngb* is also expressed in the eye and the kidney (supplementary fig. S3, Supplementary Material online). A widespread expression of Mb has also been observed in various fishes (Fraser et al. 2006; Tiedke et al. 2014). This pattern may partly be explained by the expression in smooth muscles, which, for example, surround the blood vessels (Cossins et al. 2009). In the case of Ngb, expression in the vertebrate eye has already been seen before (Schmidt et al. 2003; Bentmann et al. 2005). In addition, expression of Ngb in the kidney can be explained by its localization in endocrine tissues such as the adrenal gland, as seen before in mice (Burmester et al. 2000; Reuss et al. 2002).

### The Specific Role of GbE in the Sauropsid Retina

GbE has been found in birds (Kugelstadt et al. 2004; Hoffmann et al. 2011), the coelacanth (Schwarze and Burmester 2013), and in turtles (this study). Previous studies with chicken have shown that the expression of GbE is essentially restricted to the eye (Kugelstadt et al. 2004; Blank, Kiger, et al. 2011), which agrees with the findings in turtles (fig. 4). This shows that at least in the Sauropsida the eye-specific function is a general feature of GbE, which might have emerged early in the evolution for that purpose. However, studies in “lower” vertebrates are missing because of the restricted taxonomic distribution of the protein.

GbE protein and mRNA were detected with ISH in the cytoplasm of the RPE cells of the softshell turtle and the chicken. In contrast to the previous studies (Blank, Kiger, et al. 2011), which identified GbE in the outer segments of the photoreceptor cells, the pigments were bleached before the experiments. It also should be noted, however, that the RPE cells contain long microvilli at the apical surface enclosing the rod and cones of photoreceptor cells (Boulton and Dayhaw-Barker 2001), which makes it difficult to differentiate this layers.

The retina is one of the highest oxygen-consuming tissues in the body (Anderson 1968; Ames 1992) and the adequate supply of oxygen is essential for the normal physiological function of the retina (Yu and Cringle 2001, 2005). The RPE layer acts both as a selective barrier and vegetative regulator between the choroidal capillaries and the underlying photoreceptor cells. The RPE has essential functions to ensure the retinal integrity (Jaeger and Tasman 2012), that is, visual pigment regeneration during dark light, transport of nutrients and waste products, and renewal of the photoreceptor outer segments (Saari 2000; Boulton and Dayhaw-Barker 2001). High amounts of O_2_ must be transported from the choroid capillaries to the photoreceptors via the RPE to maintain the activity of the outer retina. Thus, the most likely function of GbE is to facilitate diffusion of O_2_ across the RPE. The reasons why GbE has been lost in different vertebrate lineages are unknown. It may be speculated, however, that certain morphological or physiological adaptations improved O_2_ supply to the retina, thereby rendered GbE unnecessary (Burmester and Hankeln 2014). One such adaptation may have been the emergence of vascularized retinae in mammals.

### Oxygen Supply in Vascular and Avascular Retinae

Because of the strict segmentation of the vertebrate retina into functionally distinct layers, O_2_ delivery heavily depends on the vascularization of the retina, which varies between species (Chase 1982). In vascularized retinae, for example, in most of the mammals, the blood supply is ensured by one choroidal blood vessel, immediately adjacent to the pigment epithelium, and by an inner retinal capillary network providing blood to the outer plexiform layer and the ganglion cell layer. Some species (i.e., guinea pig, wallaby, and turtles), however, possess an avascular retinae where the inner retinal capillaries are absent (Yu and Cringle 2001; Chase 1982).

The distribution of mitochondria in retinal layers corresponds to the regions with high O_2_ consumption. In the vascularized retinae, mitochondria are localized in inner segments of the photoreceptors, the plexiform layers, and the ganglion cells (fig. 6) (Bentmann et al. 2005; Stone et al. 2008). In the avascularized retina of the guinea pig and the rabbit, mitochondria are absent in plexiform layers. Here we show that in the retinae of both, turtle and chicken, mitochondria mainly reside in the inner segments of the photoreceptors, which agrees with the high O_2_ demand of these cells. In addition, few mitochondria were also seen in the outer plexiform layer of the turtle retina. Thus, the results confirm that the deeper layers of the chicken and turtle retinae are mostly devoid of oxygen-consuming bodies. Hence, the function of GbE in the RPE is mainly to support the photoreceptors, but not the inner layers. The deep retinal capillaries in the vascularized retinae may have evolved to support the oxidative metabolism of the inner layers, which may have led to a higher efficiency of the nerve signaling process. In these retinae, local supply of the photoreceptors and inner nerves may have been taken over by Ngb (supplementary fig. S7, Supplementary Material online) (Schmidt et al. 2003; Bentmann et al. 2005; Lechauve et al. 2013).

## Conclusion

We have demonstrated that the turtles harbor and express all eight vertebrate globin types. Most likely, each member of the globin family has its specific role in the organism. The comparative expression analyses clearly showed tissue-specific expression of Mb, Ngb, and GbE, which can easily be reconciled with their roles in O_2_ supply. The reason why each the striated muscles, the brain, and the retina has its own globin may be explained by the specific properties of each globin, which may differ in their physiological properties, for example, O_2_ affinities. This does not exclude other or multiple functions of these globins, as this has been conclusively demonstrated for Mb (Flögel et al. 2001; Hendgen-Cotta et al. 2008), and which may explain the minor expression in other tissues. The functions of the other globins, Cygb, GbX, and GbY, are more difficult to tackle because a main expression site was not observed. It must be considered that the expression domain of a specific globin may not have been identified yet, or that some globins may be expressed only in certain developmental stages (e.g., the embryo) that have not yet been covered by the current analyses.

## Supplementary Material

Supplementary figures S1–S6 and tables S1–S5 are available at Genome Biology and Evolution online (http://www.gbe.oxfordjournals.org/).

Supplementary Data
