# Supplementary material for: The Full Globin Repertoire of Turtles Provides Insights into Vertebrate Globin Evolution and Functions
Source: Genome Biol Evol. 2015 May 15;7(7):1896–913. doi: 10.1093/gbe/evv114 (PMC4524481; doi:10.1093/gbe/evv114)
Supplement: Supplementary Data [file supp_7_7_1896__index.html]

The Full Globin Repertoire of Turtles Provides Insights into Vertebrate Globin Evolution and Functions — Supplementary Data 

# The Full Globin Repertoire of Turtles Provides Insights into Vertebrate Globin Evolution and Functions

## Supplementary Data

files

- Supplementary Data - pdf file
- Supplementary Data - pdf file
