# Supplementary material for: The Full Globin Repertoire of Turtles Provides Insights into Vertebrate Globin Evolution and Functions
Source: Genome Biol Evol. 2015 May 15;7(7):1896–913. doi: 10.1093/gbe/evv114 (PMC4524481; doi:10.1093/gbe/evv114)
Supplement: Supplementary Data [file supp_evv114_Supplementary_figures.pdf]

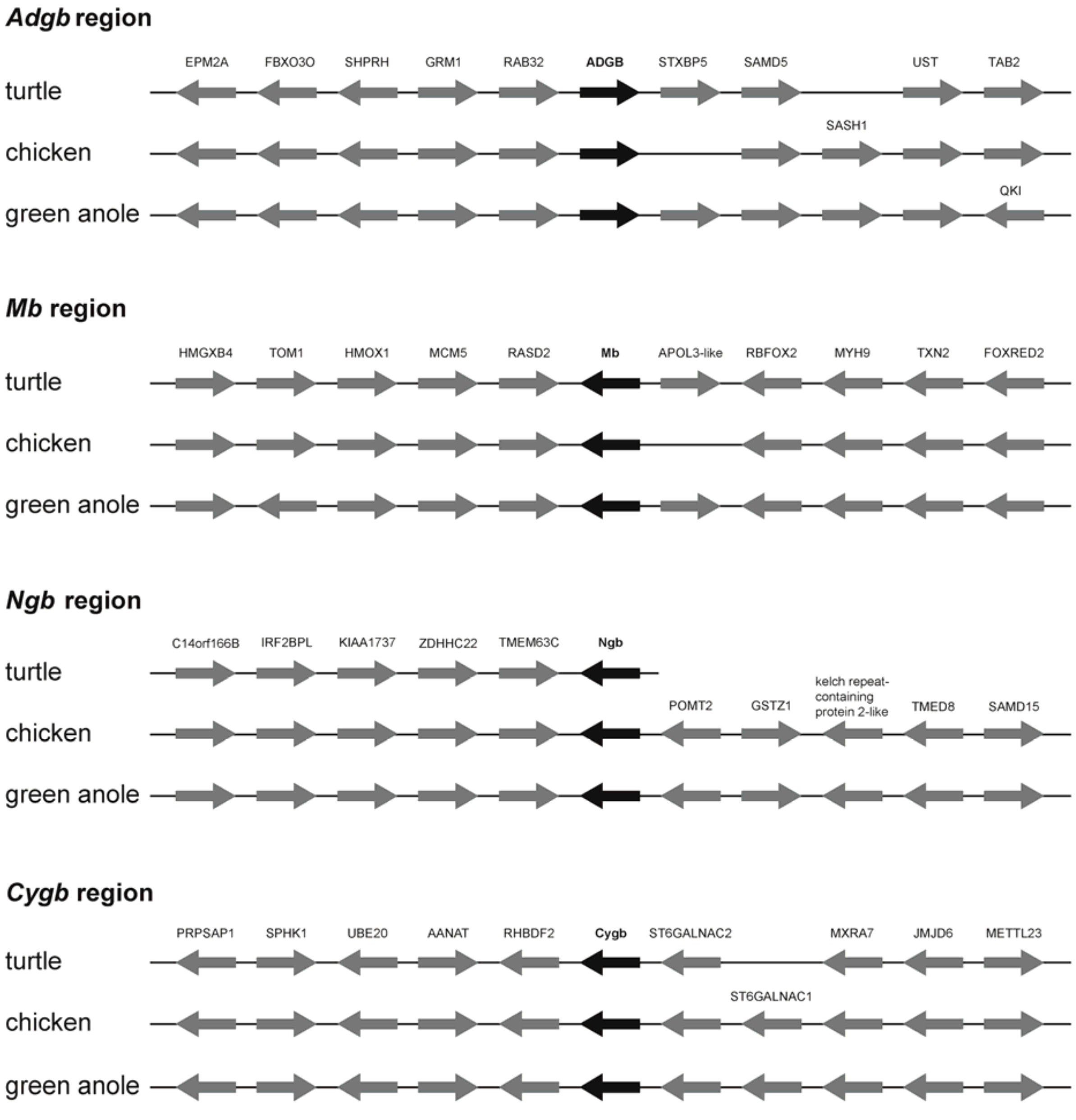

Supplementary figure S1. – Conserved synteny of the *Adgb*, *Mb*, *Ngb* and *Cygb* chromosomal regions in the genomes of turtle, chicken and *Anolis*. Globin genes are shown in black; conserved neighboring genes are shaded in dark grey. In turtles, the analysis of the Ngb region was restricted to the 3'-side of the Ngb gene due to the fragmentary assembly. The *Adgb*, *Mb*, *Ngb* and *Cygb* chromosomal regions are highly conserved.

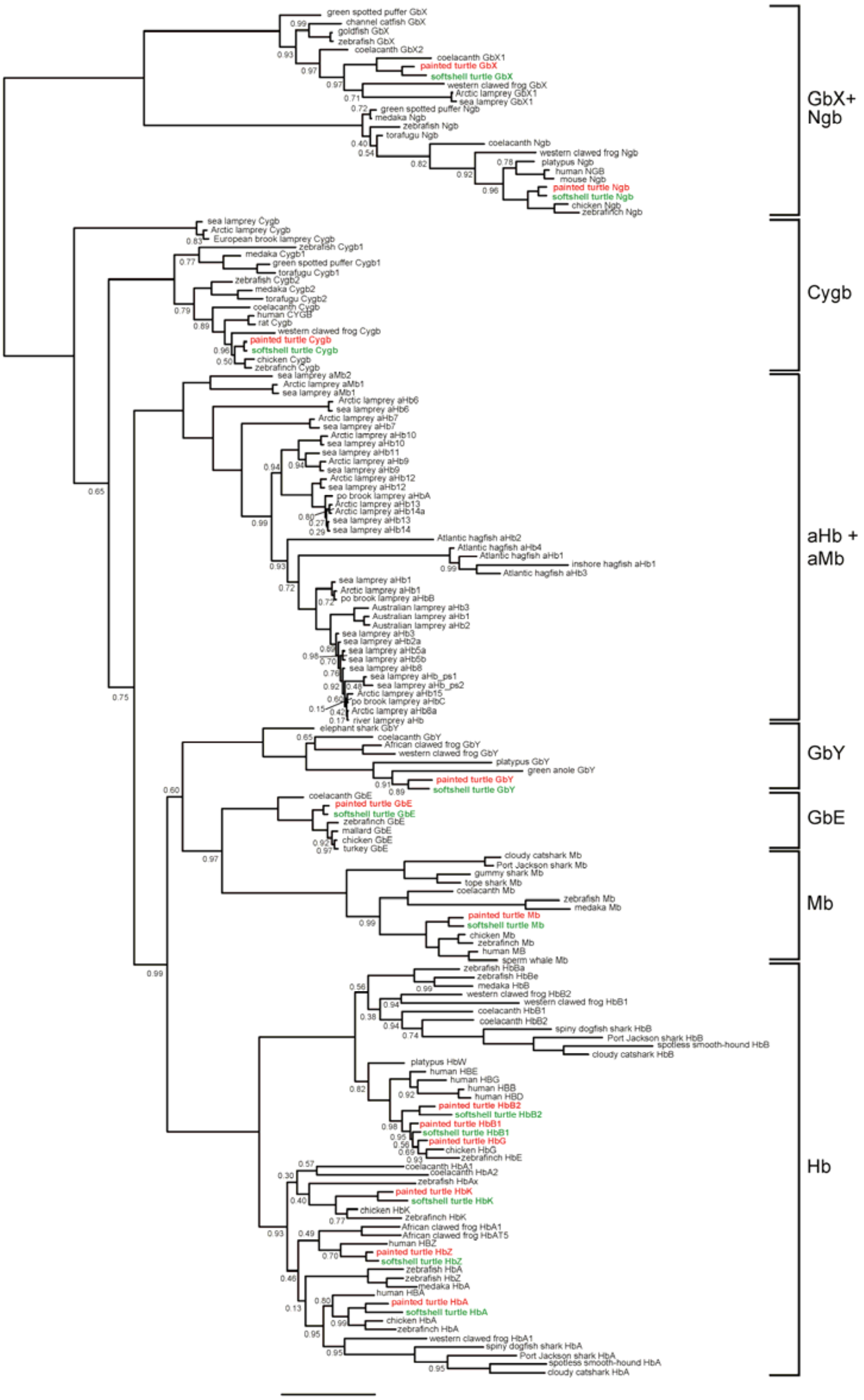

Supplementary figure S2. – Uncollapsed Bayesian phylogenetic tree of vertebrate globins. The Chinese softshell turtle globins are green; the globins of the western painted turtle globins are red. The numbers at the nodes correspond to the posterior probabilities. The bar represents 0.5 PAM distance. The common names of the species are given. aHb and aMb refer to agnathan hemoglobin and myoglobin (cf. Schwarze et al. 2014). See Supplementary Table S5 for further information on the globin proteins.

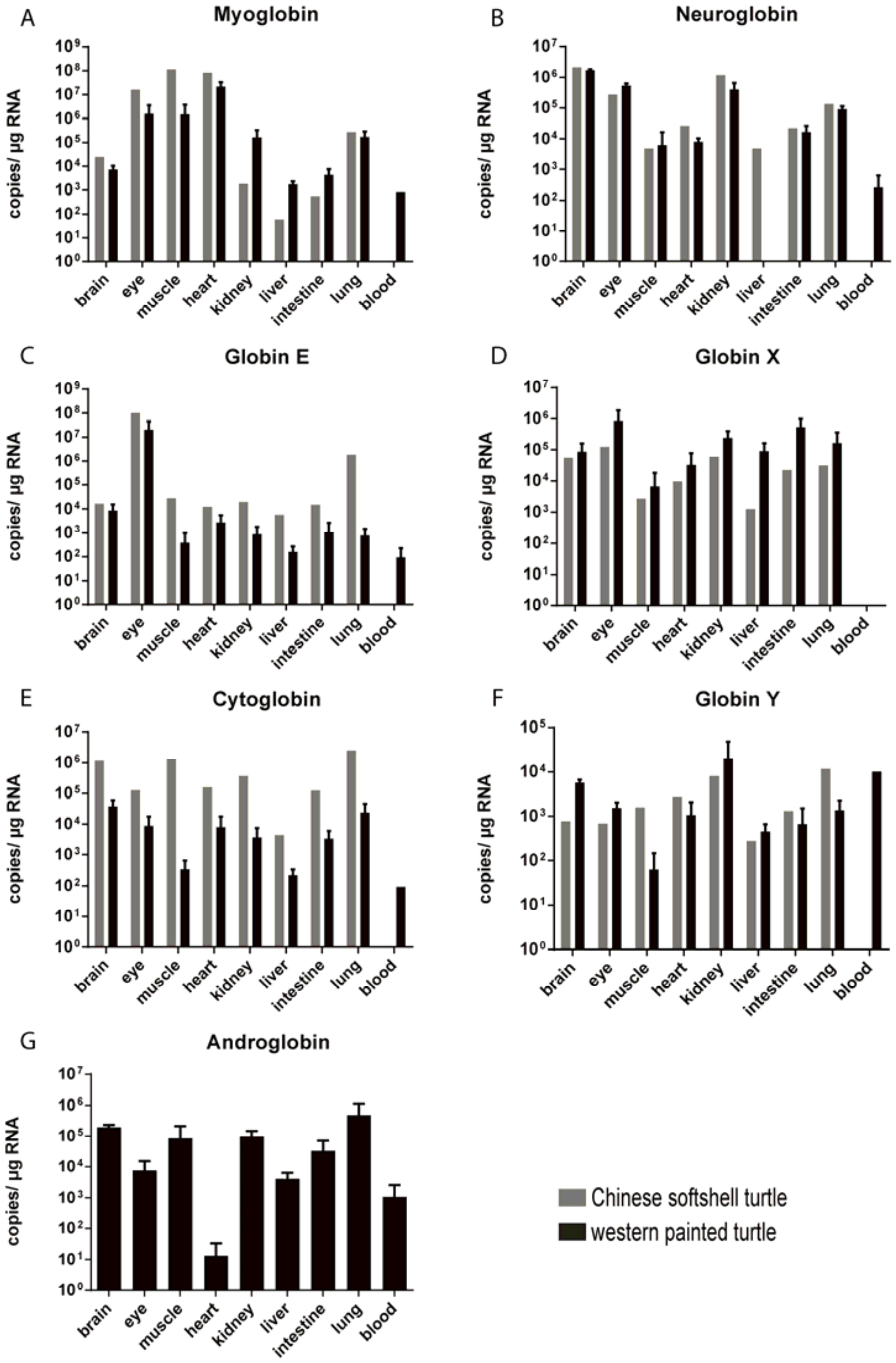

Supplementary figure S3. – Quantification of mRNA levels of vertebrate globins in different tissues of the Chinese softshell turtle (gray) and the western painted turtle (black), displayed in logarithmic scale. Absolute copy numbers of mRNA were obtained using qRT-PCR experiments. Next to the tissue specificity of *Mb* and *Ngb*, shown in fig. 4, *Mb* (A), *Ngb* (B) are also expressed in the eye, the lung and the kidney and in the eye and kidney, respectively. *GbX* (D), *Cygb* (E) and *GbY* (F) showed a widespread expression in different tissues with lower levels. Adgb (G), which was only tested in western painted turtle, is expressed in all tissues analyzed.

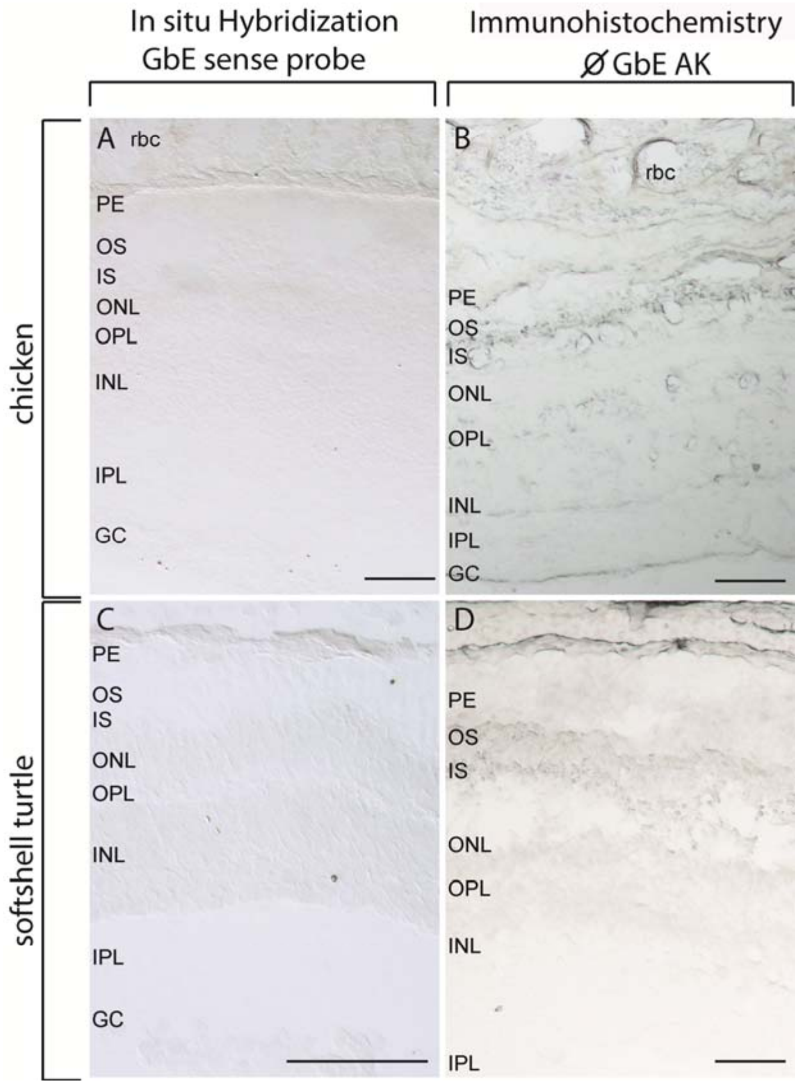

Supplementary figure S4. – Negative controls with sense probes (ISH) and omitted first antibody (IHC) of *GbE* in the retina of softshell turtle and chicken. No staining was detected. Scale bar = 100µm. rbc; red blood cells, PE: pigment epithelium, OS: outer segments of the photoreceptor cells, IS: inner segments of the photoreceptor cells, ONL: outer nuclear layer, INL: inner nuclear layer, IPL: inner plexiform layer, GC: ganglion cells.

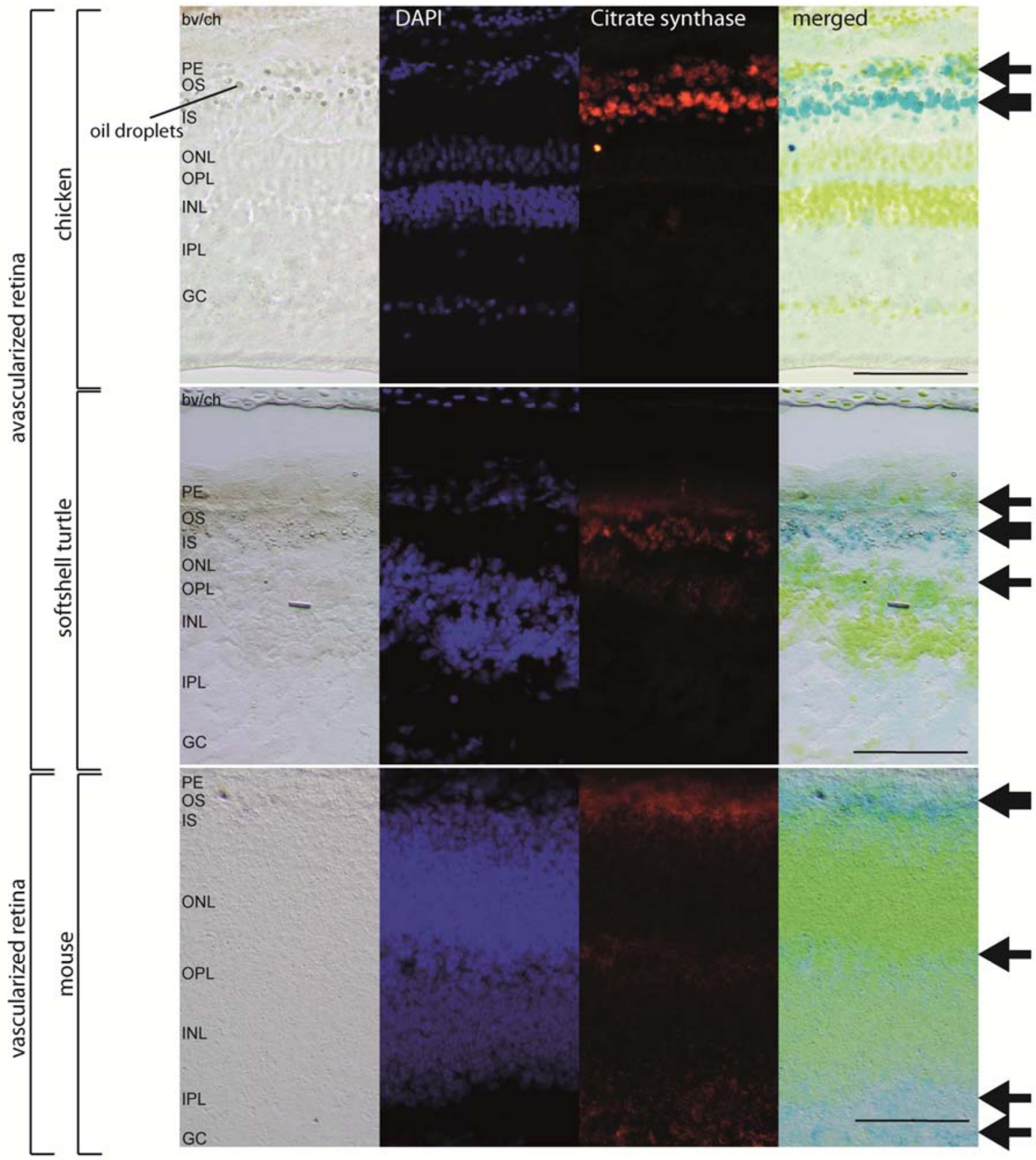

Supplementary figure S5. –Immunofluorescence of citrate synthase in avascular retinae of chicken and turtle and the vascularized retina of the mouse. The intensity of the staining is reflected by the thickness of the arrows in the different retinal layers. In chicken, the mitochondria are stained in the photoreceptor cells (OS, IS) and pigment epithelium (PE). In the softshell turtle, there is also weak staining of the outer nerve layer (ONL). The vascularized retina of the mouse showed mitochondria in the photoreceptor cells (OS, IS), the plexiform layers (IPL, OPL) and the ganglion cell layer (GC). Negative controls with omitted first antibodies are shown in supplementary fig. 6, Supplementary Material online. Scale bar = 100 µm. For abbreviations, see fig. 5.

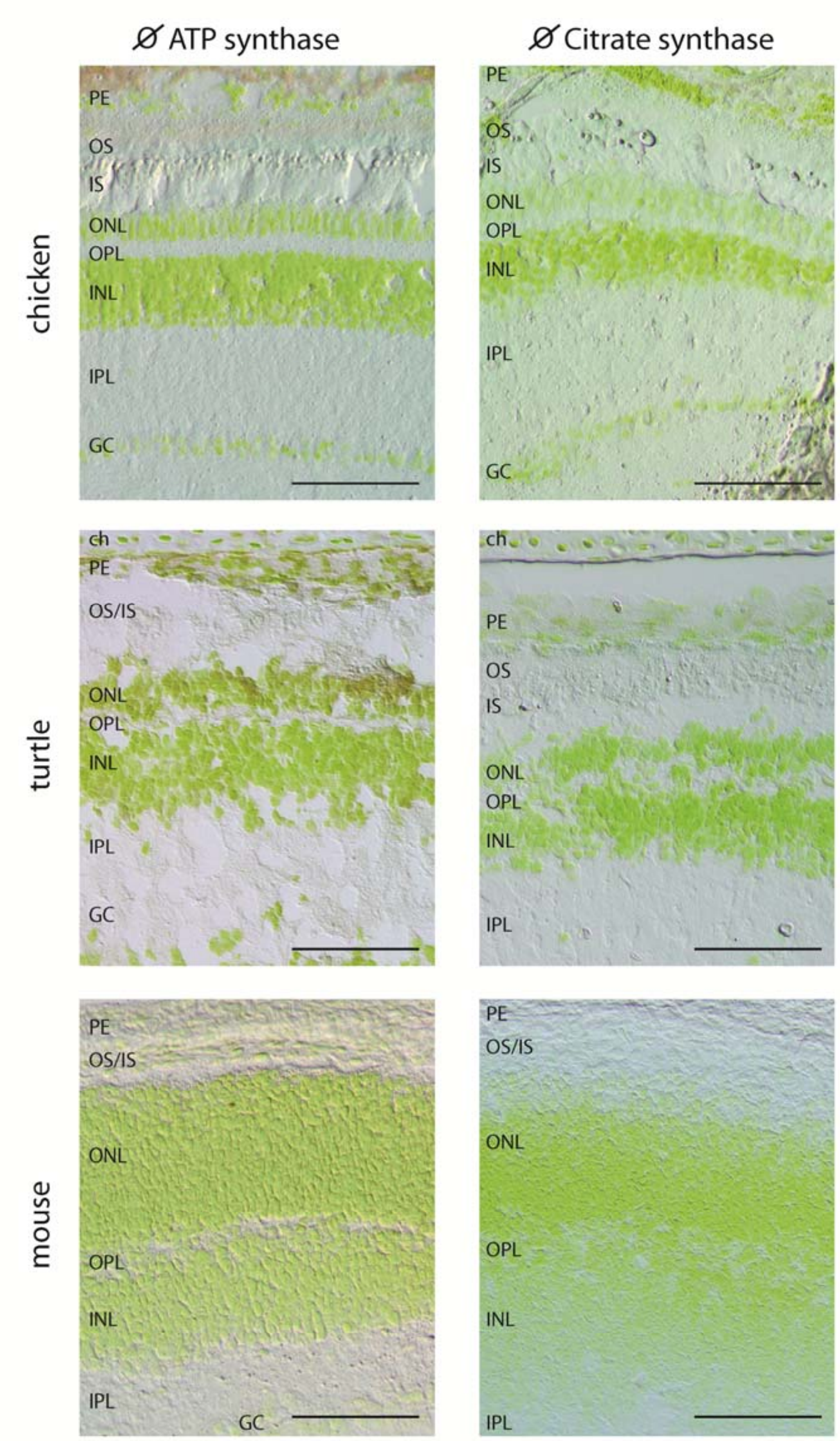

Supplementary figure S6. – Negative controls with omitted first antibody (IHC) of citrate synthase and ATP-synthase beta in avascular retinae of chicken and turtle and the vascularized retina of the mouse. No staining was detected. Nuclei are stained with Hoechst dye 33258 and displayed in yellow. Scale bar = 100 µm. ch; choroid, PE: pigment epithelium, OS: outer segments of the photoreceptor cells, IS: inner segments of the photoreceptor cells, ONL: outer nuclear layer, INL: inner nuclear layer, IPL: inner plexiform layer, GC: ganglion cells.

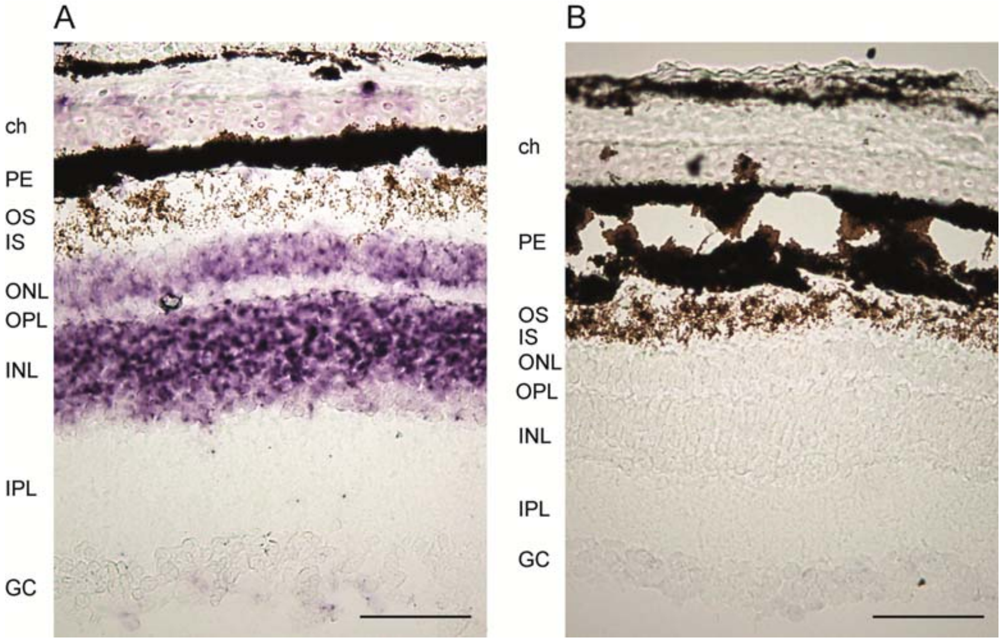

Supplementary figure S7. –Localization of *Ngb* mRNA in the retina of softshell turtle. ISH was carried out with a species-specific anti-sense probe to detect the *Ngb* mRNA (A); for negative control the sense probe was used (B). The pigments in the retina were not bleached. The *Ngb* mRNA was detected in the nuclear layers (ONL, INL). Negative control with sense probes showed no staining. Scale bar = 100µm. ch: choroid, PE: pigment epithelium, OS: outer segments of the photoreceptor cells, IS: inner segments of the photoreceptor cells, ONL: outer nuclear layer, INL: inner nuclear layer, IPL: inner plexiform layer, GC: ganglion cells.
