# Supplementary material for: The Full Globin Repertoire of Turtles Provides Insights into Vertebrate Globin Evolution and Functions
Source: Genome Biol Evol. 2015 May 15;7(7):1896–913. doi: 10.1093/gbe/evv114 (PMC4524481; doi:10.1093/gbe/evv114)
Supplement: Supplementary Data [file supp_evv114_Supplementary_tables.pdf]

Supplementary table S1. Oligonucleotides used for RT-PCR, qPCR, RACE-PCR and ISH.

Gene	Label	5'-3'sequence
**Myoglobin**	Cpi_Mb_for	TCTGGGCGAAAGTGGAAC
	Cpi_Mb_rev	ACCGAACTCCTTGTACTTGC
	Cpi_Mb_qPCR_for	ATAATCAGACTCTTTCAGGTTCATCCCGAGACC
	Cpi_Mb_qPCR_rev	TGGTGCCGTGTTTCTTCACTTCTTCG
	Psi_Mb_for	GGAAGTTATGATCAGGCTCTTTCAGGTTCAC
	Psi_Mb_rev	TTCATCTGAGCTCTTCATCTCATCCGCC
**Neuroglobin**	Cpi_Ngb_for	GATTATCCAGCACACAACAGGC
	Cpi_Ngb_rev	GCTTTCACCACGGAACCATAC
	Cpi_Ngb_qPCR_for	GGCATCGTCTTGTTTACCAGGTTGTTTGATC
	Cpi_Ngb_qPCR_rev	GATGAGAGGCACTCCTGAGGGCTG
	Psi_Ngb_for	CCTGGATCACATTAGGAAGGTGATGCTTG
	Psi_Ngb_rev	CCCCAACTGCCTGGTGTTTCTTGC
	Psi_Ngb_ISH_for	ATGGAGAGTGGAAGATTATCCAGC
	Psi_Ngb_ISH_rev	CTATTCCCCCTCTTTACGTGCA
**Cytoglobin**	Cpi_Cygb_for	TTGAGAGATGGGAAAGGAGCG
	Cpi_Cygb_rev	GGTGTAGATGAGGCTCTTC
	Cpi_Cygb_qPCR_for	ACTCCGAAAAGGTCTCATCTGTTTTGGC
	Cpi_Cygb_qPCR_rev	GGTGAAGAACTTGAAGTAGACAGGCTCCA
	Psi_Cygb_for	CACCGTAGTGGAGAACATCAATGACTCC
	Psi_Cygb_rev	GCCGGTGAAGAACTTGAAGTAGACAGG
**Globin E**	Cpi_GbE_for	CCTGGGAGAAGATGTATGCC
	Cpi_GbE_rev	GCAGATTTCGTTGGTCACCTT
	Cpi_GbE_qPCR_for	ACTGATGCTTTCCTTGGGATTGTGAACC
	Cpi_GbE_qPCR_rev	TCACAGATTATCCTAAAGTTCTTGGGGTCAACC
	Psi_GbE_for	CTGTGCTGGTCAGAATGTTTACAGAACACC
	Psi_GbE_rev	CCATGACTCCTGATCTGATCTGACTGTTCC

	Psi_GbE_ISH_for	ATGGCGTTCTCTGAAGCAGAA
	Psi_GbE_ISH_rev	CCAGCCAGCTTCTTTATAGGCA
**Globin X**	Cpi_GbX_for	ATGGGGTGCGCTCTGTCG
	Cpi_GbX_rev	CTGCTCCCTACCTGCCAGG
	Cpi_GbX_qPCR_for	GCCTGAGGGTGATGTCCTTCATTGA
	Cpi_GbX_qPCR_rev	CTTGGGAGGGGCGTTGTATCTGTAG
	Psi_GbX_for	CCTTCATCGAGAAAAGCGTGGCCC
	Psi_GbX_rev	GCCAACGTACTCATAGTACTTGGGAGG
**GlobinY**	Cpi_GbY_for	CACATCTGGGCAAAGTTATT
	Cpi_GbY_rev	GTTCAGAGAGCAGACCAAA
	Cpi_GbY_qPCR_for	TGAATTTCCAGAGCATGTTTCAGGTGATCC
	Cpi_GbY_qPCR_rev	CAGACCAAAGAGCTTCTCCCAGGAG
	Psi_GbY_for	GCATACTTCAAGAGCATCCCGACTGAAGG
	Psi_GbY_rev	CAACCATGACTCTCCGCCCATGG
**Hemoglobin**	Psi_HbK_5'rev	CAGGTTGTAGGCATGCAGGTCG
	Psi_HbK_5'rev_nested	CGTCTTGCTGAGGGTGTCCATGTG
	Psi_HbA_3'for	CCACCAAGACCTACTTCGCC
	Psi_HbA_3'for_nested	GGCAAGAAGGTGCTGAGCG

Supplementary table S2. Globin genes of the Chinese softshell turtle.

Globin genes	Accession number	Length (aa)/exons	Genomic location
Adgb	XM_006111304.1	1450/31	JH205385: 482872 - 698835
Ngb	XM_006117796.1	160/4	JH207292: 8582005-8577248
GbX	not annotated	218/5	JH211032: 890205-873421
Cygb	XM_006136486.1	179/3	JH212636:3508930-3478787
Mb	ENSPSIG00000008494	154/3	JH224648: 224623-229563
GbE	XM_006127809	151/3	JH209952: 146997- 153094
HbZ	ENSPSIG00000012161	142/3	JH209131: 354922-352105
HbK	ENSPSIG00000012157, not correctly annotated	141/3	JH209131: 342037-345748 (1st + 2nd exon), sequence was completed by RACE.
HbA	ENSPSIG00000011874; ENSPSIG00000011856	142/3	JH209131: 336263-337115 (1st + 2nd exon); 329346-330180 (1st + 2nd exon), sequences were each completed by RACE.
HbB1	ENSPSIG00000004624	147/3	JH210967: 92793-94274
HbB2	ENSPSIG00000005077	147/3	JH210967: 107525-108943
GbY	XM_006124603.1	154/3	JH209131:310883 - 315182

Supplementary table S3. Globin genes of the western painted turtle.

Globin genes	Accession number (GenBank)	Length (aa)/exons	Genomic location (ENSEMBLE)
Adgb	XM_005280422.1<br>XM_005280423.1<br>XM_005280424.1<br>XM_005280425.1<br>XM_005280426.1	1596/38	Chr 3: 42171821- 42391301 [Chrysemys_picta_bellii-3.0.3 (GCF_000241765.3)]
Ngb	XM_005301643.2	160/4	JH584728: 4213166- 4208484
GbX	XM_005293187.1	222/5	JH584610:1442038- 1451887
Cygb	XM_005297540.2	179/3	JH584685: 4299504- 4270945
Mb	XM_005300826.2	154/3	JH584171: 6110702- 6094271
GbE	XM_005293060.2	151/3	JH584608 :826847- 830855
HbZ	XM_005306148.1,<br>XM_008162535.1<br>(transcripts are identical)	142/3	JH584800: 1628402- 1631333
HbK	XM_005306157.1	141/3	JH584800: 1638554- 1639378
HbA	XM_005306158.1	142/3	JH584800: 1645419- 1646202
HbB1	XM_005290010.1	147/3	JH584561: 1328654- 1326746
HbB2	XM_005290009.1	147/3	JH584561: 1316355- 1314155
HbG	XM_005290008.1	147/3	JH584561: 1310563- 1308793
GbY	XM_005306194.2	154/3	JH584800: 1665335- 1660554

Supplementary table S4. Identities and similarities of the turtle globin proteins, and their orthologs in the chicken.

Identity [%]/ Similarity [%]		Western painted turtle													Chicken
		HbZ	HbK	HbA	HbB1	HbB2	HbG	Mb	Ngb	Cygb	GbE	GbY	GbX	Adgb	
Chinese softshell turtle	HbZ	91.5/ 94.4													81.7/ 88.0
	HbK		82.3/ 89.4												68.8/ 83.0
	HbA			76.1/ 86.6											69.0/ 82.4
	HbB1				90.5/ 98.0										85.0/ 93.9
	HbB2					81.0/ 89.1	-								75.5/ 86.4
	Mb							88.3/ 92.9							76.6/ 84.4
	Ngb								92.5/ 96.9						76.2/ 88.8
	Cygb									97.8/ 98.9					84.4/ 91.6
	GbE										96.0/ 98.7				85.4/ 94.0
	GbY											80.5/ 91.6			-
	GbX												82.0/ 90.1		-
	Adgb													88.3/ 92.6	62.9/ 74.9
Chicken		83,1/ 90,1	72.3/ 86.5	76.1/ 83.1	88.4/ 96.0	80.3/ 91.8	-	75.3/ 85.7	78.1/ 88.8	84.9/ 92.2	85.4/ 92.7	-	-	64.6/ 76.7	

Supplementary table S5. List of globin sequences used for phylogenetic analyses (fig. 3 and Supplementary fig. S1). EMBL/GenBank/ENSEMBL accession numbers of nucleotides are given. Asterisks denote protein accession numbers.

**Globin**	**Label**	**Species**	**Common name**	**Accession number**
GbX	goldfish GbX	*Carassius auratus*	goldfish	AJ635195
GbX	painted turtle GbX	*Chrysemis picta bellii*	western painted turtle	XM_005293187 + genome data
GbX	zebrafish GbX	*Danio rerio*	zebrafish	AJ635194
GbX	catfish GbX	*Ictalurus punctatus*	channel catfish	CK416201
GbX	coelacanth GbX1	*Latimeria chalumnae*	coelacanth	ENSLACG00000010727
GbX	coelacanth GbX2	*Latimeria chalumnae*	coelacanth	ENSLACG00000003954
GbX	Arctic lamprey GbX1	*Lethenteron camtschaticum*	Arctic lamprey	genome data
GbX	softshell turtle GbX	*Pelodiscus sinensis*	Chinese softshell turtle	genome data
GbX	sea lamprey GbX1	*Petromyzon marinus*	sea lamprey	ENSPMAG00000007241
GbX	green spotted puffer GbX	*Tetraodon nigroviridis*	green spotted puffer	AJ635193
GbX	western clawed frog GbX	*Xenopus tropicalis*	western clawed frog	NM_001011196
Ngb	painted turtle Ngb	*Chrysemis picta bellii*	western painted turtle	XM_005301643
Ngb	zebrafish Ngb	*Danio rerio*	zebrafish	BC059416
Ngb	chicken Ngb	*Gallus gallus*	chicken	NM_001031551
Ngb	human NGB	*Homo sapiens*	man	AB463927
Ngb	coelacanth Ngb	*Latimeria chalumnae*	coelacanth	genome data
Ngb	mouse Ngb	*Mus musculus*	mouse	AJ245945
Ngb	platypus Ngb	*Ornithorhynchus anatinus*	platypus	XM_001508367
Ngb	medaka Ngb	*Oryzias latipes*	medaka	DK002855
Ngb	softshell turtle Ngb	*Pelodiscus sinensis*	Chinese softshell turtle	ENSPSIG00000004493
Ngb	zebra finch Ngb	*Taeniopygia guttata*	zebra finch	ENSTGUG00000012286
Ngb	torafugu Ngb	*Takifugu rubripes*	torafugu	XM_003962460
Ngb	green spotted puffer Ngb	*Tetraodon nigroviridis*	green spotted puffer	AJ315609
Ngb	western clawed frog Ngb	*Xenopus tropicalis*	western clawed frog	NM_001030351
aHb	inshore hagfish aHb1	*Eptatretus burgeri*	inshore hagfish	Q7SID0*
aHb	river lamprey aHb	*Lampetra fluviatilis*	river lamprey	P02207*
aHb	Arctic lamprey aHb1	*Lethenteron camtschaticum*	Arctic lamprey	AB294236
aHb	Arctic lamprey	*Lethenteron*	Arctic lamprey	genome data

	aHb2a	*camtschaticum*		
aHb	Arctic lamprey aHb6	*Lethenteron camtschaticum*	Arctic lamprey	genome data
aHb	Arctic lamprey aHb7	*Lethenteron camtschaticum*	Arctic lamprey	AB294237
aHb	Arctic lamprey aHb9	*Lethenteron camtschaticum*	Arctic lamprey	genome data
aHb	Arctic lamprey aHb10	*Lethenteron camtschaticum*	Arctic lamprey	genome data
aHb	Arctic lamprey aHb12	*Lethenteron camtschaticum*	Arctic lamprey	genome data
aHb	Arctic lamprey aHb13	*Lethenteron camtschaticum*	Arctic lamprey	genome data
aHb	Arctic lamprey aHb14a	*Lethenteron camtschaticum*	Arctic lamprey	genome data
aHb	Arctic lamprey aHb15	*Lethenteron camtschaticum*	Arctic lamprey	genome data
aHb	Po brook lamprey aHbA	*Lethenteron zanandreai*	Po brook lamprey	Z24746
aHb	Po brook lamprey aHbB	*Lethenteron zanandreai*	Po brook lamprey	Z24747
aHb	Po brook lamprey aHbC	*Lethenteron zanandreai*	Po brook lamprey	Z24748
aHb	Australian lamprey aHb1	*Mordacia mordax*	Australian lamprey	P21197*
aHb	Australian lamprey aHb2	*Mordacia mordax*	Australian lamprey	P21198*
aHb	Australian lamprey aHb3	*Mordacia mordax*	Australian lamprey	P21199*
aHb	Atlantic hagfish aHb1	*Myxine glutinosa*	Atlantic hagfish	AF184239
aHb	Atlantic hagfish aHb2	*Myxine glutinosa*	Atlantic hagfish	AF157494
aHb	Atlantic hagfish aHb3	*Myxine glutinosa*	Atlantic hagfish	AF184047
aHb	Atlantic hagfish aHb4	*Myxine glutinosa*	Atlantic hagfish	AF156936
aHb	sea lamprey aHb1	*Petromyzon marinus*	sea lamprey	P09967*
aHb	sea lamprey aHb2a	*Petromyzon marinus*	sea lamprey	Q9I9I3*
aHb	sea lamprey aHb3	*Petromyzon marinus*	sea lamprey	P09968*
aHb	sea lamprey aHb5a	*Petromyzon marinus*	sea lamprey	P02208*
aHb	sea lamprey aHb5b	*Petromyzon marinus*	sea lamprey	ENSPMAG00000005354
aHb	sea lamprey aHb6	*Petromyzon marinus*	sea lamprey	EG333697

aHb	sea lamprey aHb7	*Petromyzon marinus*	sea lamprey	EE278870
aHb	sea lamprey aHb8	*Petromyzon marinus*	sea lamprey	ENSPMAG00000005367
aHb	sea lamprey aHb9	*Petromyzon marinus*	sea lamprey	ENSPMAG00000008540
aHb	sea lamprey aHb10	*Petromyzon marinus*	sea lamprey	FD718926
aHb	sea lamprey aHb11	*Petromyzon marinus*	sea lamprey	ENSPMAG00000001592
aHb	sea lamprey aHb12	*Petromyzon marinus*	sea lamprey	Scaffold GL477423
aHb	sea lamprey aHb13	*Petromyzon marinus*	sea lamprey	Scaffold GL477423
aHb	sea lamprey aHb14	*Petromyzon marinus*	sea lamprey	Scaffold GL477423
aHb	sea lamprey aHb1 ps	*Petromyzon marinus*	sea lamprey	Scaffold GL476413
aHb	sea lamprey aHb2 ps	*Petromyzon marinus*	sea lamprey	Scaffold GL480013
aMb	Arctic lamprey aMb1	*Lethenteron camtschaticum*	Arctic lamprey	genome data
aMb	sea lamprey aMb1	*Petromyzon marinus*	sea lamprey	ENSPMAG00000006056
aMb	sea lamprey aMb2	*Petromyzon marinus*	sea lamprey	EG021442
Cygb	painted turtle Cygb	*Chrysemis picta bellii*	western painted turtle	XM_005297540
Cygb	zebrafish Cygb1	*Danio rerio*	zebrafish	BC165894
Cygb	zebrafish Cygb2	*Danio rerio*	zebrafish	AJ635229
Cygb	chicken Cygb	*Gallus gallus*	chicken	NM_001008789
Cygb	human CYGB	*Homo sapiens*	man	AJ315162
Cygb	European brook lamprey Cygb	*Lampetra planeri*	European brook lamprey	TBA
Cygb	coelacanth Cygb	*Latimeria chalumnae*	coelacanth	ENSLACG00000014904
Cygb	Arctic lamprey Cygb	*Lethenteron camtschaticum*	Arctic lamprey	genome data
Cygb	medaka Cygb1	*Oryzias latipes*	medaka	NM_001104767
Cygb	medaka Cygb2	*Oryzias latipes*	medaka	NM_001104768
Cygb	softshell turtle Cygb	*Pelodiscus sinensis*	Chinese softshell turtle	ENSPSIG00000002766
Cygb	sea lamprey Cygb	*Petromyzon marinus*	sea lamprey	Scaffold GL478089+ GL477469
Cygb	rat Cygb	*Rattus norvegicus*	rat	NM_130744
Cygb	zebra finch Cygb	*Taeniopygia guttata*	zebra finch	XM_002195407
Cygb	torafugu Cygb1	*Takifugu rubripes*	torafugu	ENSTRUG00000012110
Cygb	torafugu Cygb2	*Takifugu rubripes*	torafugu	ENSTRUG00000008030

Cygb	green spotted puffer Cygb1	*Tetraodon nigroviridis*	green spotted puffer	AJ635230
Cygb	western clawed frog Cygb	*Xenopus tropicalis*	western clawed frog	NM_001006869
GbY	green anole GbY	*Anolis carolinensis*	green anole	ENSACAG00000027586
GbY	elephant shark GbY	*Callorhinchus milii*	elephant shark	JK861558
GbY	painted turtle GbY	*Chrysemis picta bellii*	western painted turtle	XM_005306194
GbY	coelacanth GbY	*Latimeria chalumnae*	coelacanth	XM_005990799
GbY	platypus GbY	*Ornithorhynchus anatinus*	platypus	AC203513
GbY	softshell turtle GbY	*Pelodiscus sinensis*	Chinese softshell turtle	ENSPSIG00000011829
GbY	African clawed frog GbY	*Xenopus laevis*	African clawed frog	NM_001095686
GbY	western clawed frog GbY	*Xenopus tropicalis*	western clawed frog	XM_002941194
GbE	mallard GbE	*Anas platyrhynchos*	mallard	genome data
GbE	painted turtle GbE	*Chrysemis picta bellii*	western painted turtle	XM_005293060 + genome data
GbE	chicken GbE	*Gallus gallus*	chicken	NM_001008786
GbE	coelacanth GbE	*Latimeria chalumnae*	coelacanth	ENSLACG00000001661
GbE	turkey GbE	*Meleagris gallopavo*	turkey	XM_003202331
GbE	softshell turtle GbE	*Pelodiscus sinensis*	Chinese softshell turtle	XM_006124603
GbE	zebra finch GbE	*Taeniopygia guttata*	zebra finch	XM_002196350
Mb	painted turtle Mb	*Chrysemis picta bellii*	western painted turtle	XM_005300826
Mb	zebrafish Mb	*Danio rerio*	zebrafish	AY337025
Mb	tope shark Mb	*Galeorhinus galeus*	tope shark	P14397*
Mb	chicken Mb	*Gallus gallus*	chicken	XM_003202347
Mb	Port Jackson shark Mb	*Heterodontus portusjacksoni*	Port Jackson shark	P02206*
Mb	human MB	*Homo sapiens*	man	NM_203377
Mb	coelacanth Mb	*Latimeria chalumnae*	coelacanth	ENSLACG00000007526
Mb	gummy shark Mb	*Mustelus antarcticus*	gummy shark	P14399*
Mb	medaka Mb	*Oryzias latipes*	medaka	BJ883657
Mb	softshell turtle Mb	*Pelodiscus sinensis*	Chinese softshell turtle	ENSPSIG00000008494
Mb	sperm whale Mb	*Physeter catodon*	sperm whale	AB271144
Mb	cloudy catshark Mb	*Scyliorhinus torazame*	cloudy catshark	FY417124
Mb	zebra finch Mb	*Taeniopygia guttata*	zebra finch	XM_002199380
HbA	painted turtle HbA	*Chrysemis picta bellii*	western painted turtle	XM_005306158

HbA	painted turtle HbK	*Chrysemis picta bellii*	western painted turtle	XM_005306157
HbA	painted turtle HbZ	*Chrysemis picta bellii*	western painted turtle	XM_005306148
HbA	zebrafish HbA	*Danio rerio*	zebrafish	BC164447
HbA	zebrafish HbZ	*Danio rerio*	zebrafish	AY325264
HbA	zebrafish HbAx	*Danio rerio*	zebrafish	AL915033
HbA	chicken HbA	*Gallus gallus*	chicken	NM_001004376
HbA	chicken HbK	*Gallus gallus*	chicken	XM_003643192
HbA	Port Jackson shark HbA	*Heterodontus portusjacksoni*	Port Jackson shark	P02021*
HbA	human HBA	*Homo sapiens*	man	AF105974
HbA	human HBZ	*Homo sapiens*	man	M24173
HbA	coelacanth HbA1	*Latimeria chalumnae*	coelacanth	ENSLACG00000006283
HbA	coelacanth HbA2	*Latimeria chalumnae*	coelacanth	P23740*
HbA	spotless smooth-hound HbA	*Mustelus griseus*	spotless smooth-hound	Q9YGW2*
HbA	medaka HbA	*Oryzias latipes*	medaka	BAC20295*
HbA	softshell turtle HbA	*Pelodiscus sinensis*	Chinese softshell turtle	ENSPSIG00000011874; ENSPSIG00000011856 +RACE
HbA	softshell turtle HbK	*Pelodiscus sinensis*	Chinese softshell turtle	ENSPSIG00000012157+ RACE
HbA	softshell turtle HbZ	*Pelodiscus sinensis*	Chinese softshell turtle	ENSPSIG00000012161
HbA	cloudy catshark HbA	*Scyliorhinus torazame*	cloudy catshark	FY415186
HbA	spiny dogfish HbA	*Squalus acanthias*	spiny dogfish	P07408*
HbA	zebra finch HbA	*Taeniopygia guttata*	zebra finch	DQ216719
HbA	zebra finch HbK	*Taeniopygia guttata*	zebra finch	DQ213486
HbA	African clawed frog HbA1	*Xenopus laevis*	African clawed frog	X02796
HbA	African clawed frog HbAT5	*Xenopus laevis*	African clawed frog	X02798
HbA	western clawed frog HbA1	*Xenopus tropicalis*	western clawed frog	BC088005
HbB	painted turtle HbB1	*Chrysemis picta bellii*	western painted turtle	XM_005290010
HbB	painted turtle HbB2	*Chrysemis picta bellii*	western painted turtle	XM_005290009
HbB	painted turtle HbG	*Chrysemis picta bellii*	western painted turtle	XM_005290008
HbB	zebrafish HbBa	*Danio rerio*	zebrafish	BC164283
HbB	zebrafish HbBe	*Danio rerio*	zebrafish	NM_198073
HbB	chicken HbG	*Gallus gallus*	chicken	M73995
HbB	Port Jackson	*Heterodontus*	Port Jackson shark	P02143*

	shark HbB	*portusjacksoni*		
HbB	human HBB	*Homo sapiens*	man	NM_000518
HbB	human HBD	*Homo sapiens*	man	NM_000519
HbB	human HBE	*Homo sapiens*	man	NM_005330
HbB	human HBG	*Homo sapiens*	man	BC130457
HbB	coelacanth HbB1	*Latimeria chalumnae*	coelacanth	P23741*
HbB	coelacanth HbB2	*Latimeria chalumnae*	coelacanth	XM_006011048
HbB	spotless smooth-hound HbB	*Mustelus griseus*	spotless smooth-hound	Q9YGW1*
HbB	platypus HbW	*Ornithorhynchus anatinus*	platypus	AC203513
HbB	medaka HbB	*Oryzias latipes*	medaka	AB080120
HbB	softshell turtle HbB1	*Pelodiscus sinensis*	Chinese softshell turtle	ENSPSIG00000004624
HbB	softshell turtle HbB2	*Pelodiscus sinensis*	Chinese softshell turtle	ENSPSIG00000005077
HbB	cloudy catshark HbB	*Scyliorhinus torazame*	cloudy catshark	FY415474
HbB	spiny dogfish HbB	*Squalus acanthias*	spiny dogfish	P07409*
HbB	zebra finch HbE	*Taeniopygia guttata*	zebra finch	NM_001245112
HbB	western clawed frog HbB1	*Xenopus tropicalis*	western clawed frog	NM_203528
HbB	western clawed frog HbB2	*Xenopus tropicalis*	western clawed frog	NM_001016495
